# The *Verticillium dahliae* Sho1‐MAPK pathway regulates melanin biosynthesis and is required for cotton infection

**DOI:** 10.1111/1462-2920.14846

**Published:** 2019-11-24

**Authors:** Jun‐Jiao Li, Lei Zhou, Chun‐Mei Yin, Dan‐Dan Zhang, Steven J. Klosterman, Bao‐Li Wang, Jian Song, Dan Wang, Xiao‐Ping Hu, Krishna V. Subbarao, Jie‐Yin Chen, Xiao‐Feng Dai

**Affiliations:** ^1^ Laboratory of Cotton Disease Institute of Food Science and Technology, Chinese Academy of Agricultural Sciences Beijing 100193 China; ^2^ Key Laboratory of Agro‐products Quality and Safety Control in Storage and Transport Process Ministry of Agriculture Beijing 100193 China; ^3^ Department of Plant Pathology University of California, Davis, c/o United States Agricultural Research Station Salinas California 93905 USA; ^4^ State Key Laboratory of Crop Stress Biology for Arid Areas College of Plant Protection, Northwest A&F University Yangling 712100 China; ^5^ United States Department of Agriculture Agricultural Research Service Salinas California 93905 USA

## Abstract

*Verticillium dahliae* is a soil‐borne fungus that causes vascular wilt on numerous plants worldwide. The fungus survives in the soil for up to 14 years by producing melanized microsclerotia. The protective function of melanin in abiotic stresses is well documented. Here, we found that the *V*. *dahliae* tetraspan transmembrane protein VdSho1, a homolog of the *Saccharomyces cerevisiae* Sho1, acts as an osmosensor, and is required for plant penetration and melanin biosynthesis. The deletion mutant Δ*Sho1* was incubated on a cellophane membrane substrate that mimics the plant epidermis, revealing that the penetration of Δ*Sho1* strain was reduced compared to the wild‐type strain. Furthermore, VdSho1 regulates melanin biosynthesis by a signalling mechanism requiring a kinase‐kinase signalling module of Vst50‐Vst11‐Vst7. Strains, Δ*Vst50*, Δ*Vst7* and Δ*Vst11* also displayed defective penetration and melanin production like the Δ*Sho1* strain. Defects in penetration and melanin production in Δ*Sho1* were restored by overexpression of *Vst50*, suggesting that Vst50 lies downstream of VdSho1 in the regulatory pathway governing penetration and melanin biosynthesis. Data analyses revealed that the transmembrane portion of VdSho1 was essential for both membrane penetration and melanin production. This study demonstrates that Vst50‐Vst11‐Vst7 module regulates VdSho1‐mediated plant penetration and melanin production in *V*. *dahliae*, contributing to virulence.

## Introduction

Fungal pathogens have evolved multiple strategies to attack their hosts, and surface recognition and penetration are among the most critical processes during plant infection. (Bahn *et al*., 2007). These early recognition events occur through numerous signal‐transduction systems to sense and respond to their environments. This facilitates survival and proliferation in a range of biological niches, involving membrane‐localized sensor proteins that perceive a chemical cue or physical signal from the host (such as osmosis, oxidation, hormones, etc.), and transduce these signals from the extracellular environment to cytoplasmic effectors to activate downstream signalling pathways (Bahn *et al*., 2007; Saito, 2010). The study of surface recognition by sensors and signal transduction pathways is therefore important to understand the adaptation of fungal pathogens to their hosts.

Fungi perceive and respond to a variety of dynamic signals through ubiquitous and evolutionarily conserved mitogen‐activated protein kinase (MAPK) signalling pathways, which play critical roles in many cellular processes (Gustin *et al*., 1998). As a typical eukaryotic model, the budding yeast *Saccharomyces cerevisiae* has five MAPK pathways, governed by Hog1, Slt2, Fus3, Kss1 and Smk1 respectively, that are activated by different stimuli and several MAPKs with overlapping upstream activation elements (Gustin *et al*., 1998; Saito, 2010). Following the well‐characterized MAPK pathway in *S*. *cerevisiae*, orthologues of MAPKs have been determined to play critical roles in the infection processes of other fungi (Turrà *et al*., 2014). For instance, the rice blast fungus *Magnaporthe oryzae* PMK1, part of a highly conserved MAP kinase signal transduction pathway, is essential for regulating the development of infection structure and pathogenesis (Xu and Hamer, 1996). Typically, the signalling transduction by MAP kinase cascades is delivered from the conserved membrane‐spanning proteins that serve as biosensors to activate downstream MAP kinase cascades (Turrà *et al*., 2014). Several conserved membrane‐spanning proteins that serve as sensors of cellular processes such as osmotic stress, oxidative stress, pheromones, nutrients, cell wall integrity, mating and morphological development include Sho1, Msb2, Hkr1, Opy2, Sln1 and Ste2 (Turrà *et al*., 2014; Tatebayashi *et al*., 2015; Kou and Naqvi, 2016).

The synthetic high osmolarity sensitive (Sho1) sensor shares a conserved domain architecture in fungi that generally activates the high osmolarity glycerol (HOG)‐MAPK signalling pathway in response to high osmolarity. In *S*. *cerevisiae*, the HOG pathway is initiated by two independent osmosensors, Sho1 and Sln1, that transmit the signalling through MAPK cascades (Sho1 branch requires Ste20, Cdc42, Ste11 and Ste50 factors, and Sln1 branch is a phosphorelay system consisting of Sln1, Ypd1 and Ssk1) (O'Rourke *et al*., 2002; Saito and Tatebayashi, 2004; Westfall *et al*., 2004), which eventually converge at the level of MAPKK Pbs2 and transduction to MAPK Hog1 (O'Rourke *et al*., 2002). The Sho1 branch employs two related mucin‐like transmembrane proteins Hkr1 and Msb2, which are the osmosensors of two sub‐branches of the HOG pathway (Tatebayashi *et al*., 2007; Tatebayashi *et al*., 2015). Typical Sho1 is a tetraspan transmembrane protein that contains an intracellular domain of Src Homology 3 (SH3) at the C‐terminus (Tong *et al*., 2002). These domains/motifs are important for mediating protein–protein interactions and scaffolding function for downstream MAPK signalling activation (Tatebayashi *et al*., 2015). In addition to sensing the osmotic stress, Sho1 is also involved in multiple functions in fungi such as hydrogen peroxide adaptation (Román *et al*., 2005), cell wall integrity (Román *et al*., 2005), antifungal target of drugs sensitivity (Boisnard *et al*., 2008) and morphogenesis development (O'Rourke and Herskowitz, 1998).

Orthologs of yeast Sho1 are important for virulence in fungal pathogens (Lanver *et al*., 2010; Liu *et al*., 2011; Gu *et al*., 2015; Perez‐Nadales and Di Pietro, 2015; Yu *et al*., 2016; Ren *et al*., 2019). For instance, in *Magnaporthe oryzae* and *Ustilago maydis*, Sho1 can influence virulence by regulating appressorium development (Lanver *et al*., 2010; Liu *et al*., 2011); In *Fusarium graminearum*, Sho1 signals via a downstream FgSte50‐Ste11‐Ste7 MAPK cascade to initiate conidiation, regulate pathogenicity, and mycotoxin biosynthesis, unlike its traditionally established role of osmotic stress response in *S*. *cerevisiae* (Gu *et al*., 2015). In the grey mold fungus *Botrytis cinerea*, the BcSHO1 shares some functional redundancy of BcSLN1 to regulate the pathogenesis (Ren *et al*., 2019). It is therefore apparent that the Sho1 plays a critical role in pathogenesis through the global regulation of initial signal recognition and transduction mediated by MAPK cascades (Ma and Li, 2013). However, the signal recognition function by Sho1 displays divergence in different pathogens. For instance, *F*. *graminearum* Sho1 (FgSho1) responds to cell wall integrity but not osmotic stress, suggesting that the function of FgSho1 is uncoupled from the HOG pathway, which is significantly different from what is known in *S*. *cerevisiae* (Gu *et al*., 2015).

*Verticillium dahliae* is a soil‐borne fungus causing devastating vascular wilt diseases on well over 200 plant species, including many economically important crops, such as cotton and tomato (Klosterman *et al*., 2009; Inderbitzin and Subbarao, 2014). This fungus infects host plants by directly penetrating roots and subsequently and systematically colonizing the water‐conducting xylem vessels, until the pathogen switches to the reproductive mode when it produces conidia and toxins that interfere with the water movement that eventually results in leaf wilting and plant death (Klosterman *et al*., 2009; Zhang *et al*., 2019). Thus far, dozens of genes regulating virulence in *V*. *dahliae* (Fradin and Thomma, 2006; Klimes *et al*., 2015; Zhang *et al*., 2017b) on various hosts have been identified.

Host penetration by *V*. *dahliae* requires a specialized infection structure called a hyphopodium, which develops following the sensing of extracellular signals from host plants and proper activation of the intracellular signalling pathways via various kinases and other signalling proteins, and is essential for penetration of plant roots (Zhao *et al*., 2016; Sarmiento‐Villamil *et al*., 2018a; Sarmiento‐Villamil *et al*., 2018b). The surface sensor and signal transduction studies have confirmed the role of these proteins in the pathogenesis in *V*. *dahliae*, including the kinases VdMsb (Tian *et al*., 2014), VdHog1 (Wang *et al*., 2016), VdPbs2 (Tian *et al*., 2016), VdSsk2 and VdSte11 (Yu *et al*., 2019), VdSsk1 (Zheng *et al*., 2019) and VMK1 (Tzima *et al*., 2010). For instance, deletion of the *V*. *dahliae* surface sensor gene *Msb2* (*VdMsb*) significantly decreased virulence on cotton (Tian *et al*., 2014). *Verticillium dahliae* sensors and MAPK cascades also participate in signal transduction processes leading to melanin biosynthesis (Tzima *et al*., 2010; Tian *et al*., 2014; Tian *et al*., 2016; Wang *et al*., 2016; Wang *et al*., 2018; Yu *et al*., 2019; Zheng *et al*., 2019).

Melanin is produced by a broad variety of pathogenic microorganisms and its production may be stimulated in stress responses, including bacteria, fungi and helminths (Nosanchuk and Casadevall, 2003), but unlike the well‐characterized protective role of melanin, its roles in pathogenesis are not so clear (Howard and Valent, 1996; Griffiths *et al*., 2018). Similarly, in *V*. *dahliae* melanin biosynthesis driven by sensor and MAPK cascades is correlated with pathogenicity in some studies (Tzima *et al*., 2010; Tian *et al*., 2014; Tian *et al*., 2016; Wang *et al*., 2016; Yu *et al*., 2019; Zheng *et al*., 2019). Deletion of the *V*. *dahliae* polyketide synthase VdPKS1, which is required for melanin production, reduced the virulence of *V*. *dahliae* on cotton (Zhang *et al*., 2017). In contrast, analyses of two deletion mutants of either *VdPKS1* or *VdCmr1* within the melanin biosynthetic gene cluster in *V*. *dahliae* strain VdLs.17 revealed that neither was required for full virulence on lettuce and tobacco, but were required for survival in response to UV irradiation and high‐temperature stress (Wang *et al*., 2018). Additionally, melanin accumulation was not necessary for pathogenicity of Δ*Vst1* strains of *V*. *dahliae*, which lack pigment, yet these strains are fully pathogenic on tomato and tobacco (Sarmiento‐Villamil *et al*., 2018a; Sarmiento‐Villamil *et al*., 2018b). Albino mutants or strains, which produce microsclerotia and lack typical pigmentation or that are hyaline, are commonly characterized in *V*. *dahliae*, yet are pathogenic (Daayf *et al*., 1998; Wang *et al*., 2018). Taken together, this suggests a complex interplay between signalling mechanisms controlling melanin production and virulence in *V*. *dahliae*, and that slight reductions in virulence‐associated with those strains lacking melanin production (Zhang *et al*., 2017) may be associated with reduced ability to survive in harsh environmental conditions (Wang *et al*., 2018; Fang *et al*., 2019), such as those presented during plant colonization.

The ortholog of Sho1 transmembrane sensor in *V*. *dahliae* (VdSho1) was identified previously as an important regulatory component for pathogenicity and growth (Qi *et al*., 2016.) In the current study, we investigated mechanisms of Sho1 signalling in *V*. *dahliae* with an emphasis on (i) determining the role of VdSho1 in penetration; (ii) exploration of the role of VdSho1 in growth and melanin biosynthesis; (iii) its role in the perception of signals and transduction via a downstream kinase signalling pathway in *V*. *dahliae*; and finally to uncover the mechanism by which VdSho1 regulates melanin biosynthesis and its role in the pathogenicity of *V*. *dahliae*.

## Results

### Verticillium dahliae VdSho1 *is required for penetration of cellophane membranes*

**Figure 1:**
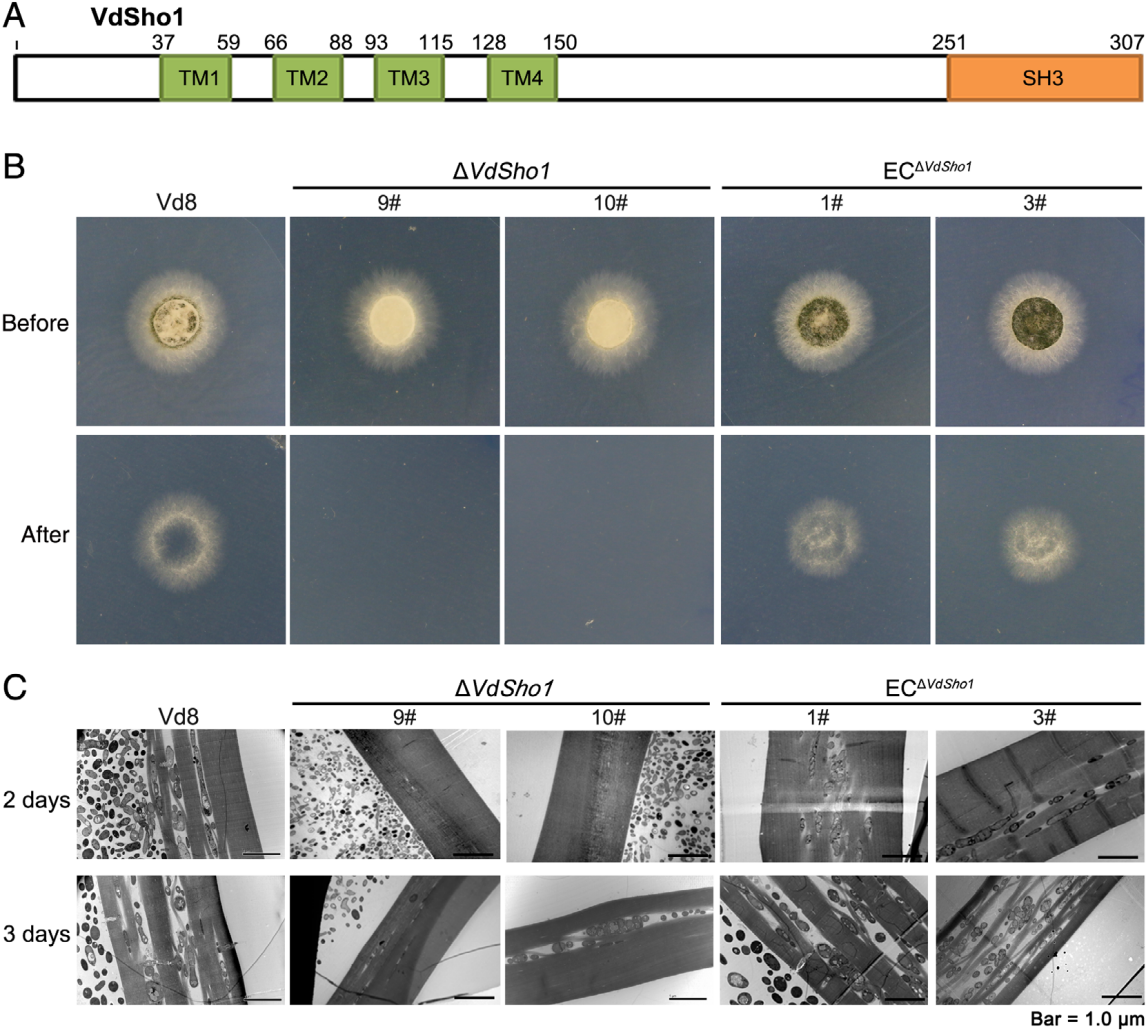
VdSho1 is required in *Verticillium dahliae* for penetration of the cellophane membrane. (A) Structure of the VdSho1 protein. Four transmembrane (TM1–TM4) motifs; SH3, Src homology 3 domain. The positions of the conserved domain or motifs were predicted using the SMART program (http://smart.embl-heidelberg.de/). (B) VdSho1 mediates cellophane membrane penetration ability in *V*. *dahliae*. Strains, including the two independent *VdSho1* gene‐deletion strains (Δ*Sho1*‐#9 and Δ*Sho1*‐#10), the two ectopic transformants (EC^Δ*Sho1*^‐#1 and EC^Δ*Sho1*^‐#3), and wild‐type strain Vd8, were grown on cellophane membranes overlaid on minimal medium (MM) for 3 days at 25°C (“Before” status). The cellophane membranes were removed from the plates and incubated for an additional 3 days to determine cellophane membrane penetration by the presence or absence of mycelial growth on the medium (“After” status). (C) Cellophane membrane penetration by *V*. *dahliae* was examined microscopically. Strain Vd8 was grown on cellophane membranes placed on MM for 2 or 3 days at 25°C. The cellophane membranes removed from the plates were analysed for penetration by transmission electron microscopy.

The *VdSho1*, was cloned from *V*. *dahliae* strain Vd8 (Zhou *et al*., 2013) according to the *V*. *dahliae* genomic sequence of strain Vd991 (Gene ID VEDA_01836; Chen *et al*., 2018). VdSho1 encodes a predicted 307 amino acid protein that contains four characteristic transmembrane domains and a C‐terminal SH3 domain in an arrangement that is well conserved in fungi (Fig. 1A; Fig. S1). To determine the role of *VdSho1* in *V*. *dahliae*, *VdSho1* deletion strains (Δ*Sho1*) and ectopic *VdSho1*‐complemented transformants were generated (Fig. S2).

Penetration analysis of the Δ*Sho1* strains on cellophane membranes revealed that the Δ*Sho1* strains were incapable of penetrating the membrane following hyphal growth on minimal medium (MM) for 3 days beyond the initial incubation period on top of the cellophane membrane (Fig. 1B). *VdSho1* complementation mutants were restored with their ability to penetrate cellophane membranes similar to the strain Vd8 (Fig. 1B). Conidia of strain Vd8 germinate and produce infectious hyphae that penetrate cellophane membranes within 2–3 days (Fig. 1C; Fig. S3). Incubation of the cellophane membranes with Δ*Sho1* strains revealed a lack of penetration across the membrane, but penetration was restored following the reintroduction of *VdSho1* into Δ*Sho1* strains (Fig. 1C). *VdSho1*, therefore, appears to be indispensable for penetration of the cellophane membrane in *V*. *dahliae*.

### *VdSho1 regulates the expression of oxidation‐related genes involved in melanin biosynthesis*

**Figure 2:**
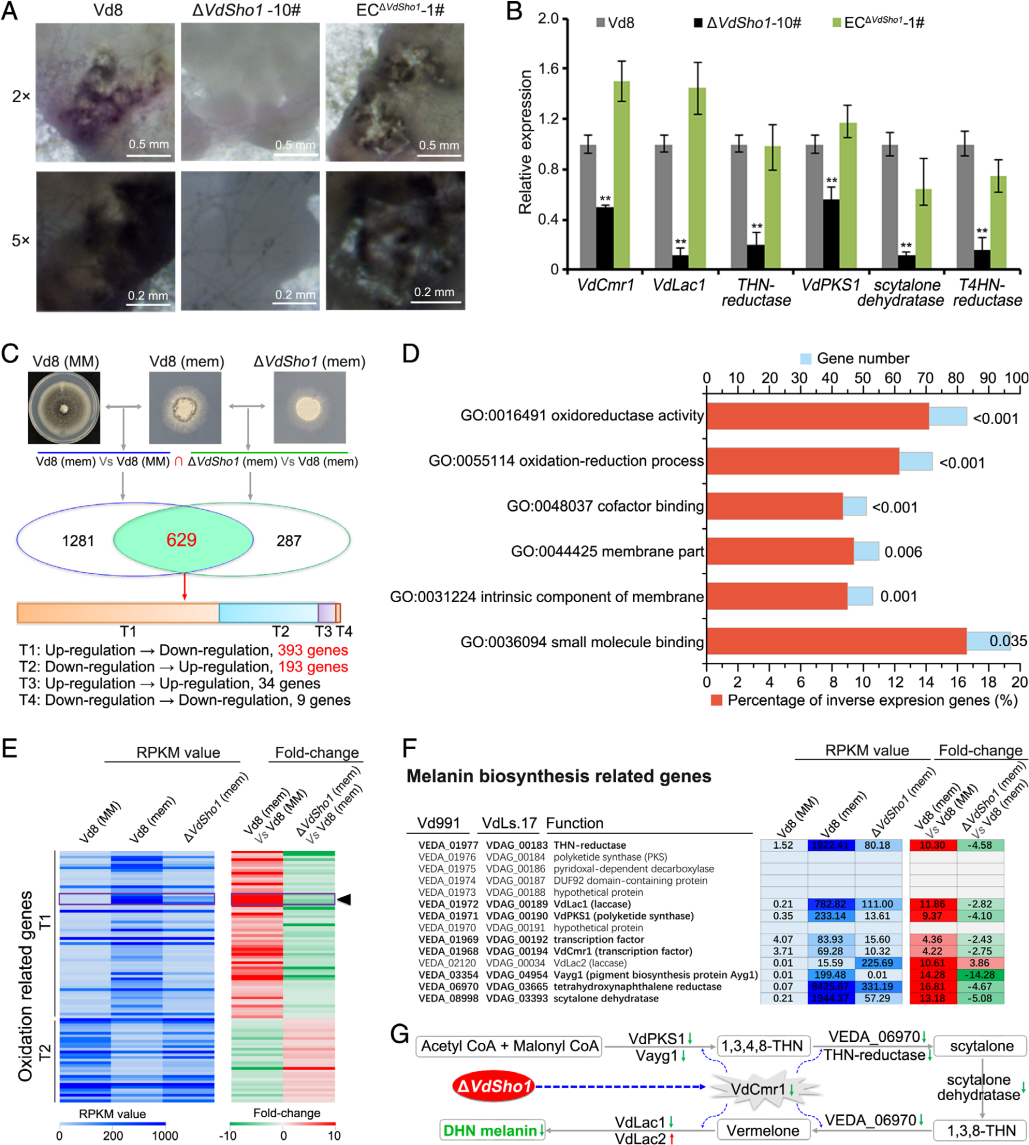
VdSho1 modulates the expression of melanin biosynthetic pathway genes in *Verticillium dahliae*. (A) Melanin accumulation affected by VdSho1 during cellophane membrane penetration. The wild‐type strain Vd8, *VdSho1* gene‐deletion strain (Δ*Sho1*‐#10) and ectopic transformant (EC^Δ*Sho1*^‐#1) were grown on cellophane membranes placed on MM medium and the phenotypes were photographed by stereomicroscope after 3 days incubation at 25°C. (B) RT‐qPCR analyses of the expression of melanin biosynthesis‐related genes regulated by VdSho1 during cellophane membrane penetration. The samples were harvested from the wild‐type strain Vd8 (set as control), *VdSho1* gene‐deletion strain (Δ*Sho1*‐#10) and ectopic transformant (EC^Δ*Sho1*^‐#1) grown on cellophane membranes placed on MM medium, the *V*. *dahliae β‐tubulin* (*VDAG_10074*) was used as an endogenous control for gene expression analysis. Error bars represent the standard deviation of three replicate experiments, and double asterisks indicates statistical significance (*P* ≤ 0.01) based on unpaired Student's *t*‐test. (C) VdSho1‐responsive genes during cellophane membrane penetration. Experimental layout for response genes mediated by VdSho1 was called through the intersection differential expressed genes (DEGs) of two comparison groups, group ellipse of the wild‐type strain Vd8 grown on cellophane membrane (Vd8(mem)) relative to that grown only on MM medium (Vd8(MM)), and green ellipse of the Δ*Sho1* strain grown on cellophane membranes (*ΔSho1* (mem)) relative to (Vd8(mem)). All the samples were harvested 3 days after incubation at 25°C. T1–T4 represent four types of expression patterns either inverse (T1 and T2) or enhanced (T3 and T4) from Vd8(mem) *Vs* Vd8(MM)) to *ΔSho1* (mem) *Vs* (Vd8(mem). (D) Significant catalogues of gene ontology (GO) enrichment of DEGs regulated by VdSho1. The significant differences (*P* < 0.05) of GO enrichment were analysed by the hypergeometric test for each GO term and subsequently corrected for multiple testing errors. (E) View of the expression data of oxidation‐related genes with inverse type expression patterns T1 and T2. The Reads per Kilobase per Million mapped reads method (RPKM) value of each sample and fold‐change value of the two comparison groups for each set of genes that were present are shown in the coloured bar. The purple box indicated by the black triangle represents the genes involved in melanin biosynthesis. (F) RPKM and fold‐change value of known genes involved in melanin biosynthesis. (G) Schematic diagram of melanin biosynthesis‐related genes regulated by VdSho1. The downward arrow in green and the upward arrow in red represent the genes down‐ or up‐regulated by VdSho1 respectively.

Interestingly, the strain Vd8 lost the ability to produce or accumulate melanin in Δ*Sho1* strains, and melanin accumulation was restored after complementation of the Δ*Sho1* strains (Fig. 1B). In the Δ*Sho1* strain, a reduction in melanin accumulation was apparent but was restored in the *VdSho1* complemented strains (Fig. 2A). RT‐qPCR analysis confirmed that six melanin synthesis‐related genes significantly regulated by VdSho1 (Fig. 2B). This suggested that penetration was accompanied by melanin accumulation in *V*. *dahliae*. To examine this correlation further, genes regulated by VdSho1 during cellophane membrane penetration were determined by RNA‐seq (|log_2_Ratio| ≥ 1.0, FDR < 0.001).

In total, 1910 genes were activated when the wild‐type strain Vd8 was grown on the cellophane membrane relative to its growth on the MM, while 916 genes were differentially expressed when both the Δ*Sho1* strain and the wild‐type strain Vd8 were grown on the cellophane membrane (Fig. 2C), suggesting a total of 629 genes specifically are regulated by VdSho1 (Fig. 2C; Table S1). Of these genes, 586 (93.2%) were positively regulated by VdSho1, displaying an inverse expression pattern (T1 and T2) relative to strain Vd8 in response to the cellophane membrane (Fig. 2C; Table S1). Gene ontology (GO) enrichment showed that the genes regulated by VdSho1 were significantly enriched in oxidation environment (*P* ≤ 0.001) (Fig. 2D), and the transcript levels (RPKM value) in GO terms (GO:0016491 and GO:0055114) were significantly up‐regulated on the cellophane membrane but was down‐regulated in the Δ*Sho1* strain (Fig. 2E). Some of the oxidation‐related genes, involved in melanin biosynthesis, were significantly regulated by VdSho1 (~10‐folds) in addition to those involved in melanin biosynthesis (Fig. 2F), and were down‐regulated following deletion of *VdSho1*, including the transcription of *VdCmr1*, *VdLac1* (laccase), and *VdPKS1* (Fig. 2G). These results suggested that gene expression associated with melanin biosynthesis is correlated with VdSho1‐mediated penetration of cellophane membranes.

### *Melanin biosynthesis plays a critical role in the penetration of* V. dahliae *into cellophane membranes*

**Figure 3:**
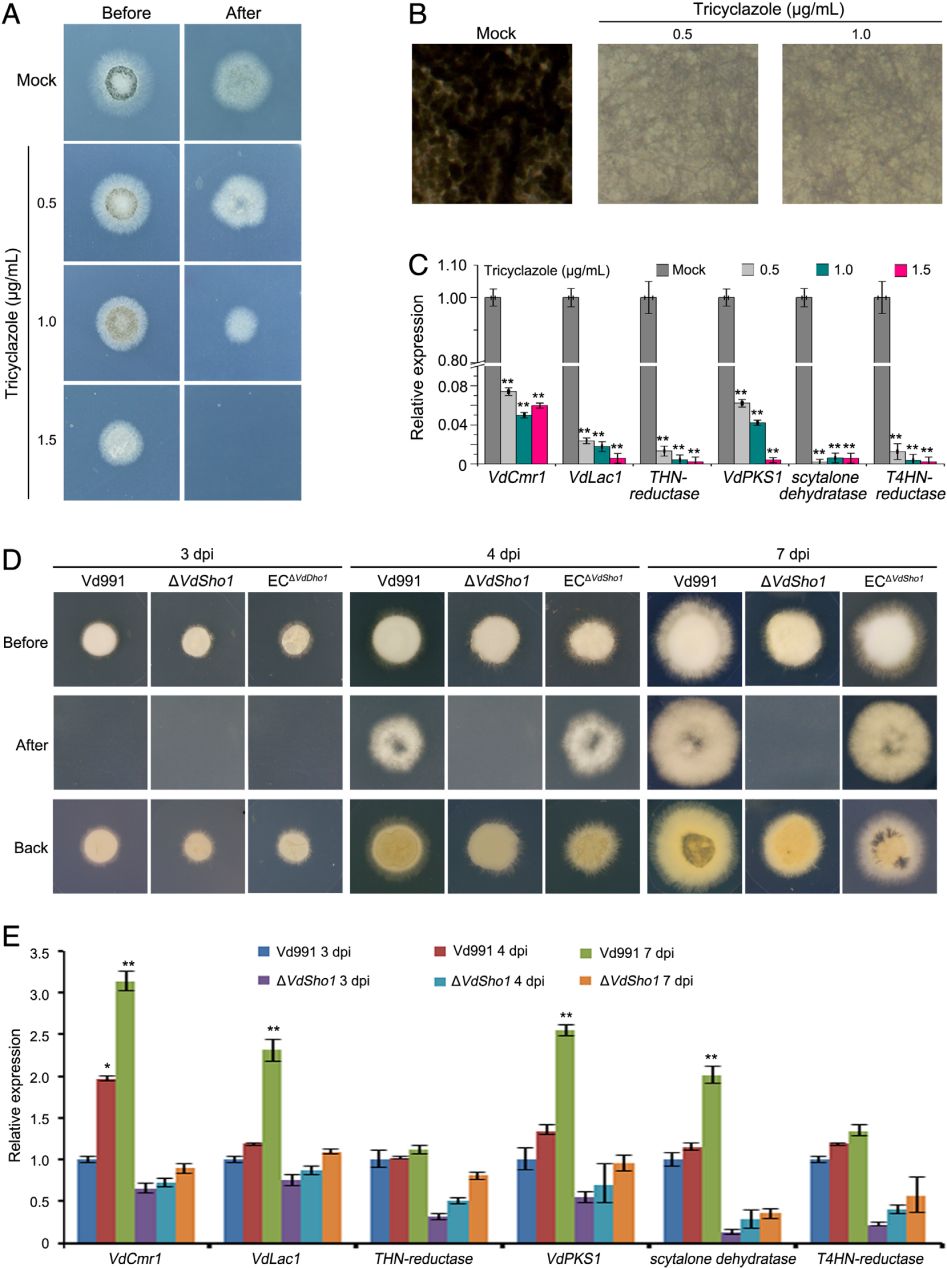
Melanin accumulation is correlated with the penetration of cellophane membranes in *Verticillium dahliae*. (A) Inhibition of the melanin biosynthesis by tricyclazole represses cellophane membrane penetration in *V*. *dahliae*. The wild‐type strain (Vd8) was grown on the cellophane membrane placed on minimal medium (MM) with the melanin biosynthesis inhibitor of tricyclazole for 3 days at 25°C incubator (set as “Before” status). The cellophane membranes were removed from the plates and continuously incubated for another 3 days to determine the cellophane membrane penetration of *V*. *dahliae* strains, by the indication of mycelium growth on medium (set as “After” status). The strain grown on the purify MM medium was set as control (Mock). (B) Investigation of the melanin accumulation by treatment with tricyclazole. The Vd8 strain was grown on top of cellophane membranes on MM medium with the tricyclazole, the phenotype were photographed by stereomicroscope 3 days after incubation at 25°C. (C) Gene expression analysis of melanin biosynthesis‐related genes response to tricyclazole. The Vd8 strain was grown on top of cellophane membranes on MM medium with the tricyclazole and the tissue was harvested at 3 days after incubation at 25°C. RT‐qPCR was performed to determine the expression levels of melanin biosynthesis‐related genes on the MM medium treatment with tricyclazole relative to growth on the purify medium (Mock). The *V*. *dahliae β‐tubulin* (*VDAG_10074*) was used as an endogenous control for gene expression analysis. Error bars represent the standard deviation of three replicate experiments and double asterisks indicate statistical significance (*P* ≤ 0.01) determined by an unpaired Student's *t*‐test. (D) VdSho1 mediates cellophane membrane penetration in hyphal type *V*. *dahliae* strain Vd991. The gene‐deletion strain and ectopic transformants of VdSho1 under the background of hyphal type strain Vd991 were grown on the top of cellophane membranes overlaid onto MM for 3, 4 and 7 days at 25°C (“Before” status). The cellophane membranes were removed from the plates and incubated for an additional 3 days to determine the cellophane membrane penetration by the presence or absence of mycelial growth on medium (“After” status). (E) RT‐qPCR analyses the expression of melanin biosynthesis‐related genes regulated by VdSho1 during cellophane membrane penetration in hyphal type *V*. *dahliae* strain Vd991. The samples were harvested from the wild‐type strain Vd991 (set as control), the gene‐deletion strain and ectopic transformant that grown on top of cellophane membranes on MM medium for 3, 4 and 7 days, the *V*. *dahliae β‐tubulin* (VDAG_10074) was used as an endogenous control. Error bars represent the standard deviation of three replicate experiments and double asterisks indicate statistical significance (*P* ≤ 0.01) determined by an unpaired Student's *t*‐test.

To further examine the potential role of melanin during penetration of cellophane membranes, we examined the effect of suppressing melanin biosynthesis on membrane penetration. Melanin accumulation was significantly reduced when grown on top of the cellophane membrane with the inhibitor tricyclazole for 3 days (Fig. 3A and B). This also correlated with reduced penetration ability as determined by hyphal growth on the MM after 3 additional days of incubation (Fig. 3A). The addition of 1.5 μg ml^−1^ tricyclazole resulted in the loss of the ability of strain Vd8 to penetrate the membrane (Fig. 3A). RT‐qPCR analysis revealed that six melanin biosynthesis‐related genes were significantly suppressed by the tricyclazole treatment (Fig. 3C). The melanin biosynthesis inhibitor carpropamid also reduced the penetration ability while simultaneously suppressing the expression of melanin biosynthesis‐related gene and melanin accumulation (Fig. S4). Further analysis revealed that ability was correlated with the melanin accumulation in four other strains, and in each case accompanied by reductions in the expression of melanin biosynthesis‐related genes and melanin accumulation in *V*. *dahliae* (Fig. S5). The type strain Vd991, which displays normal growth but lacks melanin, showed reduced ability to penetrate the cellophane membrane (Fig. S6). However, strain Vd991 exhibited melanin accumulation when grown on the top of cellophane membranes for 7 days and displayed an ability to penetrate the membrane similar to the Vd8 strain (Fig. S6). Deletion of *VdSho1* in strain Vd991 resulted in loss of penetration ability for 7 days following incubation on cellophane, and penetration ability and melanin production were restored, along with melanin accumulation after mutant complementation of *VdSho1* in the strain Vd991 (Fig. 3C and D). In addition, deletion of the *VdPKS1* in the melanin biosynthesis gene cluster resulted in suppressing melanin biosynthesis and accumulation, and the ability to penetrate cellophane membrane (Fig. S7). These results suggested that melanin accumulation is tightly correlated with the penetration of cellophane membranes in *V*. *dahliae*.

### *VdSho1 interacts physically with the central conserved region of Vst50*

**Figure 4:**
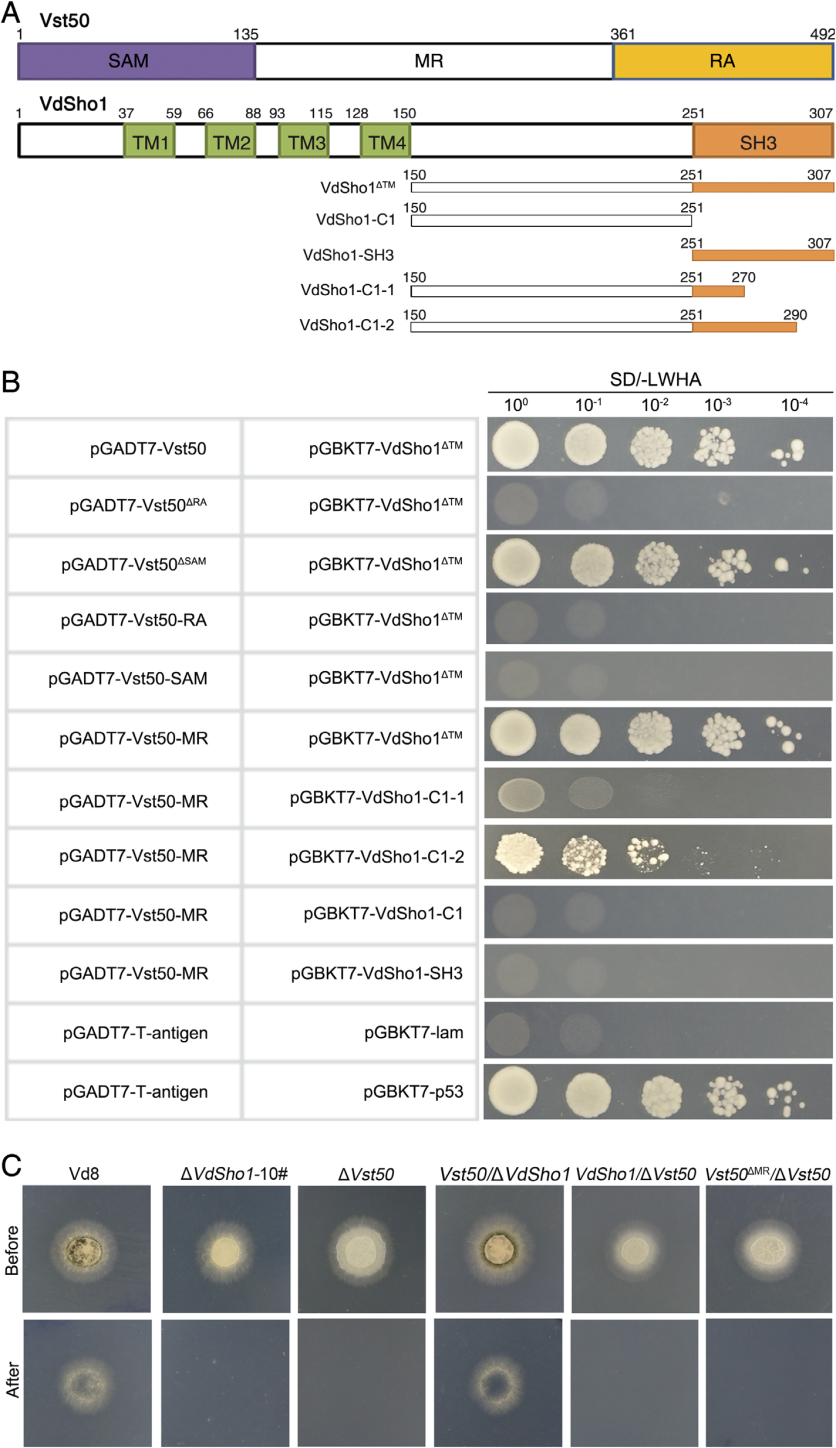
The intracellular SH3‐region of VdSho1 physically interacts with the Vst50 adapter protein. (A) Schematic map of the Vst50 and VdSho1 truncated proteins used for yeast two‐hybrid assays. Vst50 represents the *Verticillium dahliae* orthologue of Ste50 in *Saccharomyces cerevisiae*. The conserved domains of Vst50 were predicted by using SMART (http://smart.embl-heidelberg.de/); SAM represents the sterile alpha motif domain, RA represents the Ras‐associated domain, MR represents the sequence between SAM domain and RA domain. VdSho1^ΔTM^, VdSho1‐C1, VdSho1‐SH3, VdSho1‐C1‐1 and VdSho1‐C1‐2 represent the variation at the C‐terminus by VdSho1 truncations. The numbers represent the position of each in the VdSho1 and Vst50. (B) Identification of the physical interactions between VdSho1 and Vst50 by yeast two‐hybrid assays. The truncations of VdSho1 were used as bait after insertion into the pGBKT7 vector; the Vst50 and its truncations, including Vst50^ΔSAM^ (deletion of SAM domain), Vst50^ΔRA^ (deletion of RA domain), Vst50‐SAM (retention of the SAM domain), Vst50‐RA (retention of the RA domain) and Vst50‐MR (retention of the MR domain) were used as prey to after insertion of each of these cDNAs into the pGADT7 vector. Ten‐fold serially diluted yeast strains were simultaneously cultured on SD (‐Leu/‐Trp/‐His/‐Ade) plates, where only bait‐prey positive interaction strains should grow. The interaction of murine p53 (p53) and SV40 large T‐antigen (T) was used as a positive control for the system, and human lamin C (lam) was used as a negative control. (C) *Vst50* can rescue the defective cellophane membrane penetration of the Δ*Sho1* strain. The Δ*Sho1* strains (Δ*Sho1*‐#10), *Vst50* gene‐deletion strains (Δ*Vst50*), *Vst50/*Δ*Sho1* (Vst50 into Δ*Sho1* strain), *VdSho1/ΔVst50* (*VdSho1* transformed into the *V*. *dahliae* Δ*Vst50* strain), *Vst50* ^*ΔMR*^ */ΔVst50* (introduced the chimeric sequence of delete MR sequence in Vst50 into Δ*Vst50* strain), and wild‐type strain Vd8, were grown on the top of cellophane membranes on minimal medium (MM) for 3 days at 25°C incubation (“Before” status). The cellophane membranes were removed from the plates and incubated for another three days to determine cellophane membrane penetration by mycelial growth on medium (“After” status).

The MAPK cascade acts downstream of the membrane‐localized Sho1 sensor in fungi, and Ste50 plays a key role in the MAPK cascade as an adaptor or scaffold (Seet and Pawson, 2004). To determine whether VdSho1 regulates penetration of the cellophane membrane via the Vst50, the ortholog of Ste50 in *V*. *dahliae* (Vst50), adaptor involved in MAPK signalling, the interaction between VdSho1 and Vst50 was assessed by yeast two‐hybrid (YTH) system. As expected, truncation of the *N*‐terminus containing the transmembrane domain in VdSho1 allows it to physically interact with Vst50 (Fig. 4A and B). Correspondingly, the deletion of *Vst50* (Δ*Vst50*) results in the loss of ability to penetrate cellophane (Fig. 4C) and is accompanied by reduced melanin accumulation and transcript levels of melanin biosynthesis‐related genes (Fig. S8a and Fig. S8b). RNA‐seq analysis showed that 761 genes were regulated by Vst50 during cellophane penetration, and 461 (60.6%) of these were common to those regulated by VdSho1 (Fig. S8c; Table S1). The transcript levels of each gene regulated by VdSho1 and Vst50 displayed similar patterns of up‐ or down‐regulation and similar ranges of fold‐change differences in expression (Fig. S8d). The genes involved in the melanin biosynthesis pathway were also significantly reduced in the Δ*Vst50* strain (Fig. S8e). These results suggested that VdSho1 and Vst50 interact physically to modulate the penetration of cellophane and melanin biosynthesis.

The ectopic transformant of wild‐type *Vst50* into the Δ*Sho1* strain restored the ability of the strain to penetrate cellophane, similar to the strain Vd8 (Fig. 4C). In contrast, the introduction of the wild‐type *VdSho1* by ectopic transformation to the Δ*Vst50* strain failed to restore the penetration of cellophane (Fig. 4C). RT‐qPCR analysis confirmed that the transcript level of *Vst50* was significantly down‐regulated in the Δ*Sho1* strain (Fig. S9a). Similarly, the transcript levels of *VdSho1* were significantly up‐regulated in the transformant in which *VdSho1* was introduced into the Δ*Vst50* strain (Fig. S9b). There was also an increase in transcript levels of melanin biosynthesis‐related genes following the introduction of *Vst50* to the *VdSho1* deletion strain (Fig. S8f). These results suggested that Vst50 acts as the down‐stream factor of VdSho1 to affect membrane penetration and melanin production.

Sequence analysis revealed that Vst50 contains three conserved domains of the *N*‐terminal sterile alpha motif (SAM), a C‐terminal Ras‐associated domain (RA) and a domain of unknown function between SAM and RA domains (hereafter referred to as MR) (Fig. 4A). YTH assays revealed an interaction between VdSho1 and Vst50, and that this interaction occurs through the MR domain of Vst50 (Fig. 4B). Truncation of *VdSho1* and its expression in the YTH system showed that the cytoplasmic SH3 region of VdSho1 is necessary for physical interaction with the MR domain of Vst50 (Fig. 4B). Furthermore, the re‐introduction of the *Vst50* chimeric gene with the deletion of the MR domain‐encoding region into the Δ*Vst50* strain failed to restore the ability to penetrate the cellophane membrane or expression levels of the melanin biosynthesis‐related genes (Fig. 4C; Fig. S8f). Together, these results suggested that the membrane‐localized sensor VdSho1 modulates penetration through physical interaction with the MR domain of kinase scaffold protein Vst50 via its cytoplasmic SH3 domain region.

### *VdSho1 signals via a MAPK pathway to regulate penetration*

**Figure 5:**
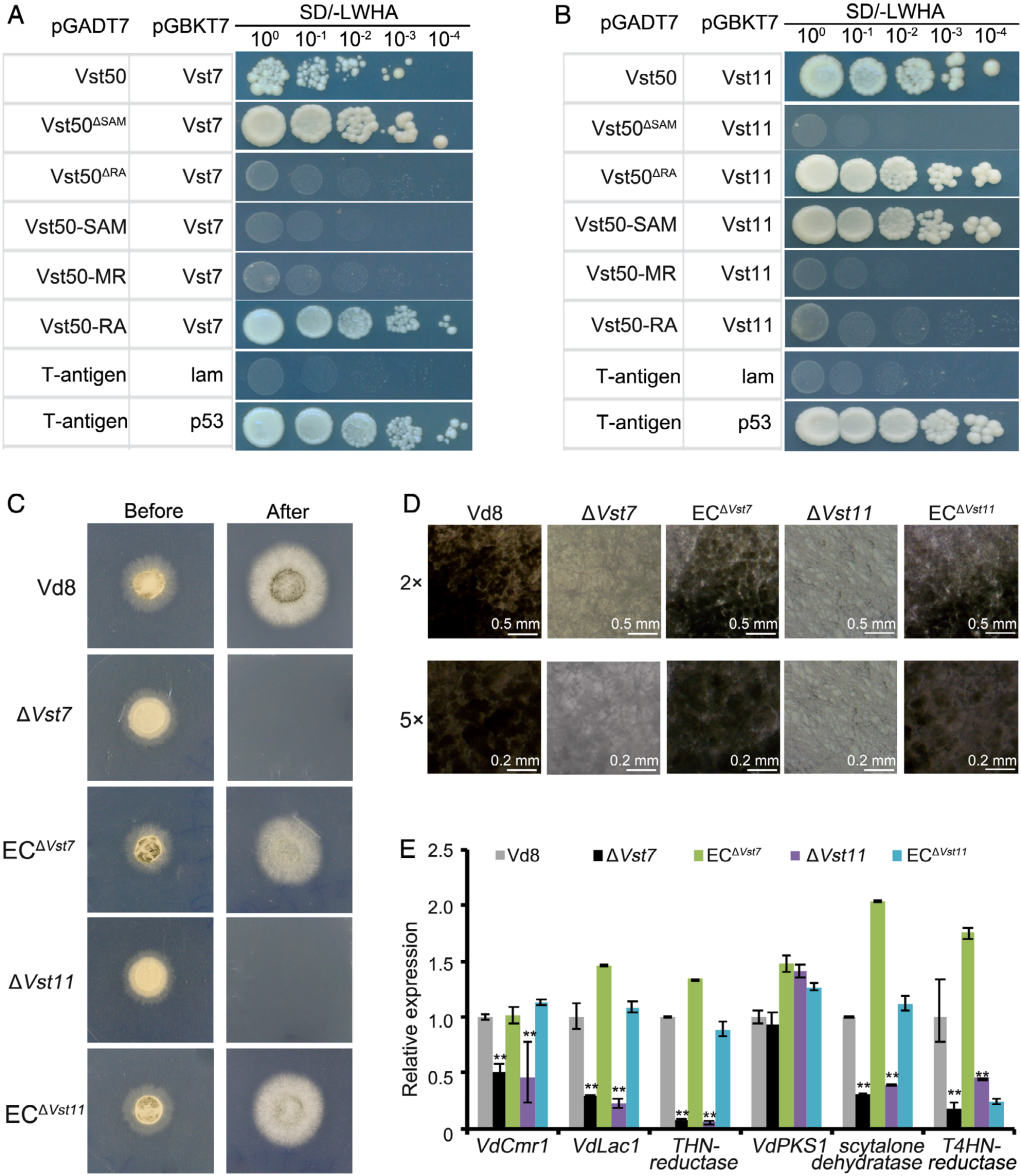
VdSho1 regulates cellophane membrane penetration and melanin accumulation by the MAPK module Vst50‐Vst7‐Vst11 in *Verticillium dahliae*. Identification of the physical interaction of Vst50 with Vst7 (A) or Vst11 (B) by yeast two‐hybrid assays. The full length coding sequences of *Vst7* and *Vst11* were inserted into the pGBKT7 bait vector. Vst50 and its truncated variants indicated in Fig. 3B were introduced into the pGADT7 prey vector. Ten‐fold serially diluted yeast strains were simultaneously cultured on SD (‐Leu/‐Trp/‐His/‐Ade) plates, where protein–protein positive interactions are selected for growth. The interaction of murine p53 (p53) and SV40 large T‐antigen (T) was used as a positive control for the system, and the interaction between T and human lamin C (lam) was a negative control reaction. (C) Identification of the cellophane membrane penetration ability mediated by Vst11 and Vst7. Strains, including *Vst11* deletion strain (Δ*Vst11*), complemented ectopic transformant (EC^Δ*Vst11*^), *Vst7* deletion strain (Δ*Vst7*), complemented ectopic transformant (EC^Δ*Vst7*^) and the wild‐type strain Vd8, were grown on the top of cellophane membranes on minimal medium (MM) for 3 days at 25°C (“Before” status). The cellophane membranes were removed from the plates and incubated for another 3 days to determine cellophane membrane penetration by presence or absence of mycelial growth (“After” status). (D) Investigation of melanin accumulation regulated by Vst11 and Vst7. Strains were grown on top of cellophane membranes on MM medium and photographed under microscopy after 3 days of incubation at 25°C. (E) Reverse transcription‐quantitative PCR (RT‐qPCR) analyses the expression of melanin biosynthesis‐related genes regulated by Vst11 or Vst7 after inoculation of strains onto cellophane membranes for penetration tests. Strains were grown on the top of cellophane membranes on MM for 3 days at 25°C. Samples were harvested for the transcript level analysis by RT‐qPCR of melanin biosynthesis‐related genes. *β‐tubulin* (*VDAG_10074*) was used as an endogenous control for gene expression analysis. Error bars represent the standard deviation of three replicate experiments and double asterisks indicate statistical significance (*P* ≤ 0.01) determined by an unpaired Student's *t*‐test.

The interaction between VdSho1 and the component of MAPK module was further assayed by YTH assay. Unlike with Vst50, VdSho1 cannot physically interact with Vst7 (ortholog of Ste7), Vst11 (ortholog of Ste11), Vst20 (ortholog of Ste20), VdVmk1 (ortholog of Vmk1) and VdCdc42 (ortholog of Cdc42) (Fig. S10a). Among the components of this MAPK cascade in *V*. *dahliae*, Vst7 and Vst11 physically interact with the RA domain and SAM domain in Vst50 (Fig. 5A and B) respectively. Furthermore, the Vst7 displayed the ability to interact with Vst11 by the YTH assay (Fig. S10b). These results suggest that VdSho1 physically interacts with Vst50 and likely signals through Vst50, which acts as a scaffold protein for the VdSho1‐MAPK module in *V*. *dahliae*.

To further probe the MAPK pathway associated with VdSho1 signalling in *V*. *dahliae*, Δ*Vst7* and Δ*Vst11* strains were generated and their complementary transformants were prepared for functional characterization of penetration and their ability to accumulate melanin or penetrate cellophane. As expected, the deletion of either *Vst7* or *Vst11* repressed their ability to penetrate cellophane, and cellophane penetration phenotype was recovered after re‐introducing *Vst7* or *Vst11* to the corresponding mutants (Fig. 5C). Both *Vst7* and *Vst11* are required for the melanin accumulation and regulate the expression of the melanin biosynthesis‐related genes (Fig. 5D and E). Re‐introduction of the chimeric gene for deletion RA or SAM domain into the *Vst50* deletion strain failed to restore the ability to penetrate or increase the expression levels of melanin biosynthetic‐related genes (Fig. S11). Additionally, genes encoding components of the MAPK cascade, including *Vst50*, *Vst7* and *Vst11*, were significantly down‐regulated in the Δ*Sho1* strains compared to the strain Vd8 at 3 days after incubation on cellophane (Fig. S12). These results further confirm that the Vst50‐Vst7‐Vst11 cascade is required for VdSho1 to mediate cellophane penetration and melanin biosynthesis under the conditions tested.

### *VdSho1 senses membrane permeabilization to regulate penetration*

**Figure 6:**
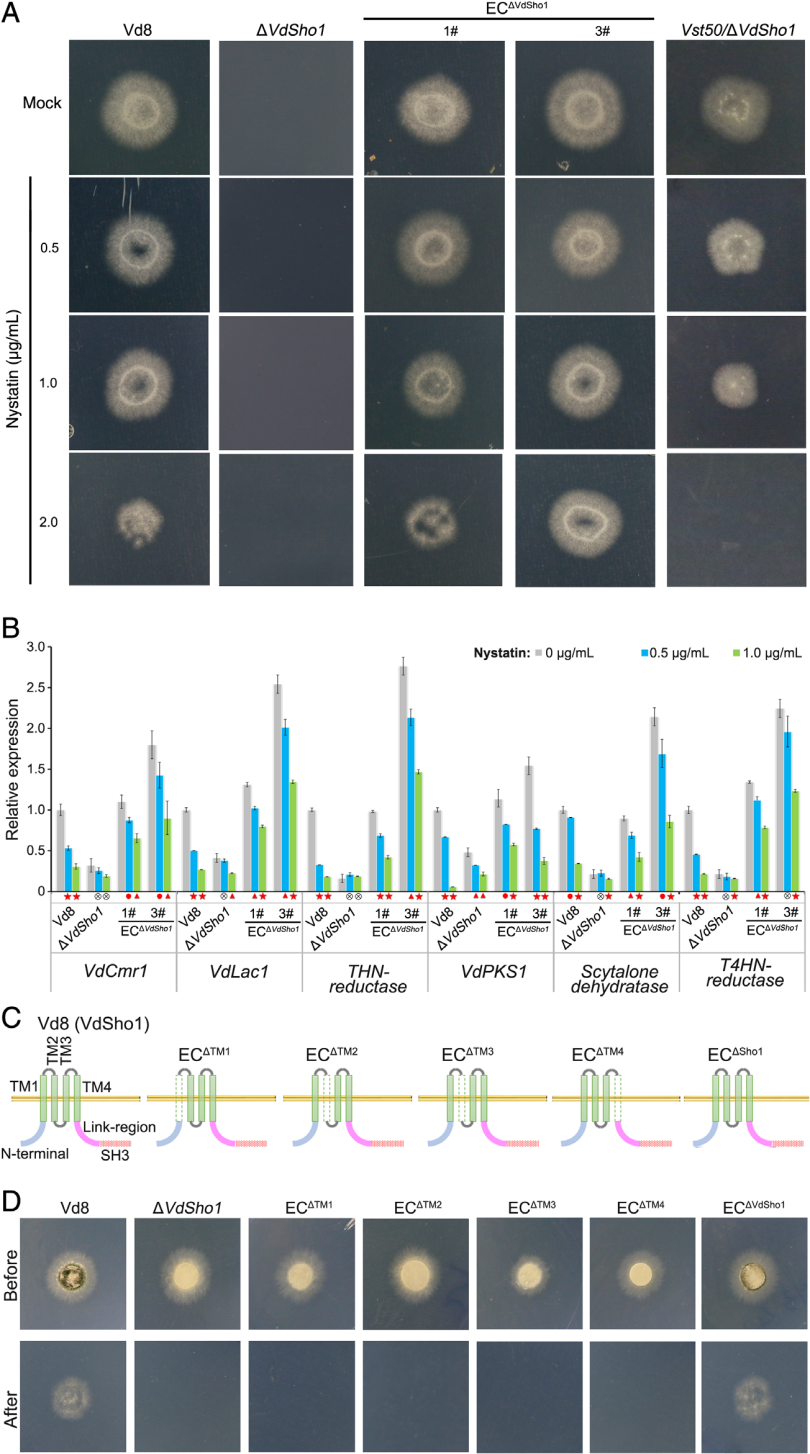
VdSho1 senses membrane permeabilization to regulate melanin biosynthesis correlated with penetration, independent of its transmembrane domains. (A) Cellophane membrane penetration is repressed by nystatin in *V*. *dahliae*. The Δ*Sho1* strain (Δ*Sho1*), two complementary transformants (EC^Δ*Sho1*^‐#1 and EC^Δ*Sho1*^‐#3), and the wild‐type strain Vd8 were grown on the top of cellophane membranes on MM with the gradient concentration (0.5, 1.0 and 2.0 μg mL^−1^) of antifungal reagent nystatin for 3 days at 25°C. Cellophane membranes were removed from the plates and the plates were incubated for another 3 days to determine the cellophane membrane penetration of *V*. *dahliae* strains by the presence or absence of mycelium growth after the additional incubation period. Pure MM medium without nystatin was used as a control (Mock). (B) Gene expression analysis of melanin biosynthesis‐related genes response to nystatin. The indicated strains were grown on the top of cellophane membranes overlaid on MM for 3 days at 25°C and the samples were harvested for the transcripts level analysis of melanin biosynthesis‐related genes. The *V*. *dahliae β‐tubulin* (*VDAG_10074*) was used as an endogenous control for gene expression analysis. Bars in grey, blue and green colour represent the indicated strains were grown with 0, 0.5 and 1.0 μg mL^−1^ nystatin respectively. Error bars represent the standard deviation of three replicate experiments, red star, red dot, and circle with cross line indicate statistical significance *P* ≤ 0.001, *P* ≤ 0.01, and *P* ≤ 0.05 determined by an unpaired Student's *t*‐test respectively. (C) Schematic map of VdSho1 domain mutations for the complementation transformations. EC^ΔTM1^, EC^ΔTM2^, EC^ΔTM3^ and EC^ΔTM4^ represent different *VdSho1* deletion mutants of each of the four transmembrane domains; EC^ΔSH3^ represents the variant of *VdSho1* having the deletion of the SH3 domain coding region. (D) Rescue of the cellophane membrane penetration ability after re‐introduction of the individual domain‐encoding region constructs of *VdSho1* into the *VdSho1* deletion strain. All the indicated strains were grown on the top of cellophane membranes on MM for 3 days at 25°C (“Before” status). Cellophane membranes were removed from the plates and the plates were incubated for an additional 3 days at 25°C to determine the cellophane membrane penetration by presence or absence of mycelial growth on medium after this additional incubation period (“After” status).

Sho1 acts as a membrane‐localized sensor that responds to multiple signals such as osmotic stress or cell wall integrity (Gu *et al*., 2015; Tatebayashi *et al*., 2015). However, the precise nature of this signalling is unclear, especially in *V*. *dahliae*. In this study, the potential signals sensed by VdSho1 were detected under the condition of high osmolarity and cell wall integrity. Unexpectedly, like the wild‐type strain Vd8, the Δ*Sho1* strain showed no significant sensitivity to the osmotic stress caused by the concentrations of NaCl or sorbitol applied, nor were there noticeable differences in the cell wall integrity in response to congo red or calcofluor white (Fig. S13 and S14), suggesting that *VdSho1* was not involved in responses to high osmotic stress and cell wall integrity in *V*. *dahliae*. Furthermore, VdSho1 sensing of the osmotic stress was determined using the pharmacological agent nystatin, which inhibits the Sho1‐mediated signalling and reduce osmotic stress intracellularly by enhancing membrane permeability (Reiser *et al*., 2003; dos Santos *et al*., 2017). In the *VdSho1* complemented transformants, the growth of Δ*Sho1* strain was not restricted in response to nystatin (Fig. S15). Furthermore, the strain Vd8 and complementary transformants displayed sensitivity to increasing concentrations of nystatin (Fig. 6A), and this phenomenon cosegregated with the transcript level of *VdSho1* in two complemented transformants. One of these (EC^ΔSho1^‐3#) that showed low sensitivity to nystatin also displayed significantly higher expression levels of *VdSho1* (Fig. 6A; Fig. S16). The introduction of *Vst50* into the Δ*Sho1* strain partially restored its sensitivity to nystatin, compromising normal growth and cellophane penetration (Fig. 6A). The expression levels of melanin biosynthesis‐related genes also were significantly regulated only in strains where the wild‐type VdSho1 was present (Fig. 6B). These findings indicated that membrane permeability regulated by nystatin suppresses the interaction between VdSho1 and Vst50, including that required for cellophane penetration. Thus, VdSho1 senses membrane permeability generated by nystatin to regulate cellophane penetration.

Previous studies have shown that Sho1 forms planar oligomers of the dimers‐of‐trimers architecture by the four transmembrane domains to sense signals (Tatebayashi *et al*., 2015). To confirm that the transmembrane domain in VdSho1 is involved in a similar sensory function, transmembrane deletion strains were constructed, and a cellophane penetration assay was conducted (Fig. 6C). The deletion of any transmembrane domain regions encoded by *VdSho1* caused a defect in the ability to penetrate the cellophane membrane (Fig. 6D). Interestingly, this defect was also associated with reduced transcript levels of melanin biosynthesis‐related genes (Fig. 6D; Fig. S17). The deletion of any transmembrane domain also abolished the ability to regulate the expression level of melanin biosynthesis‐related genes in response to nystatin (Fig. S18). These results suggested that the sensory structure is comprised of four transmembrane domains, each of which is required for the functioning of VdSho1 in penetration.

### *VdSho1 is required for full virulence of* V. dahliae *on cotton*

**Figure 7:**
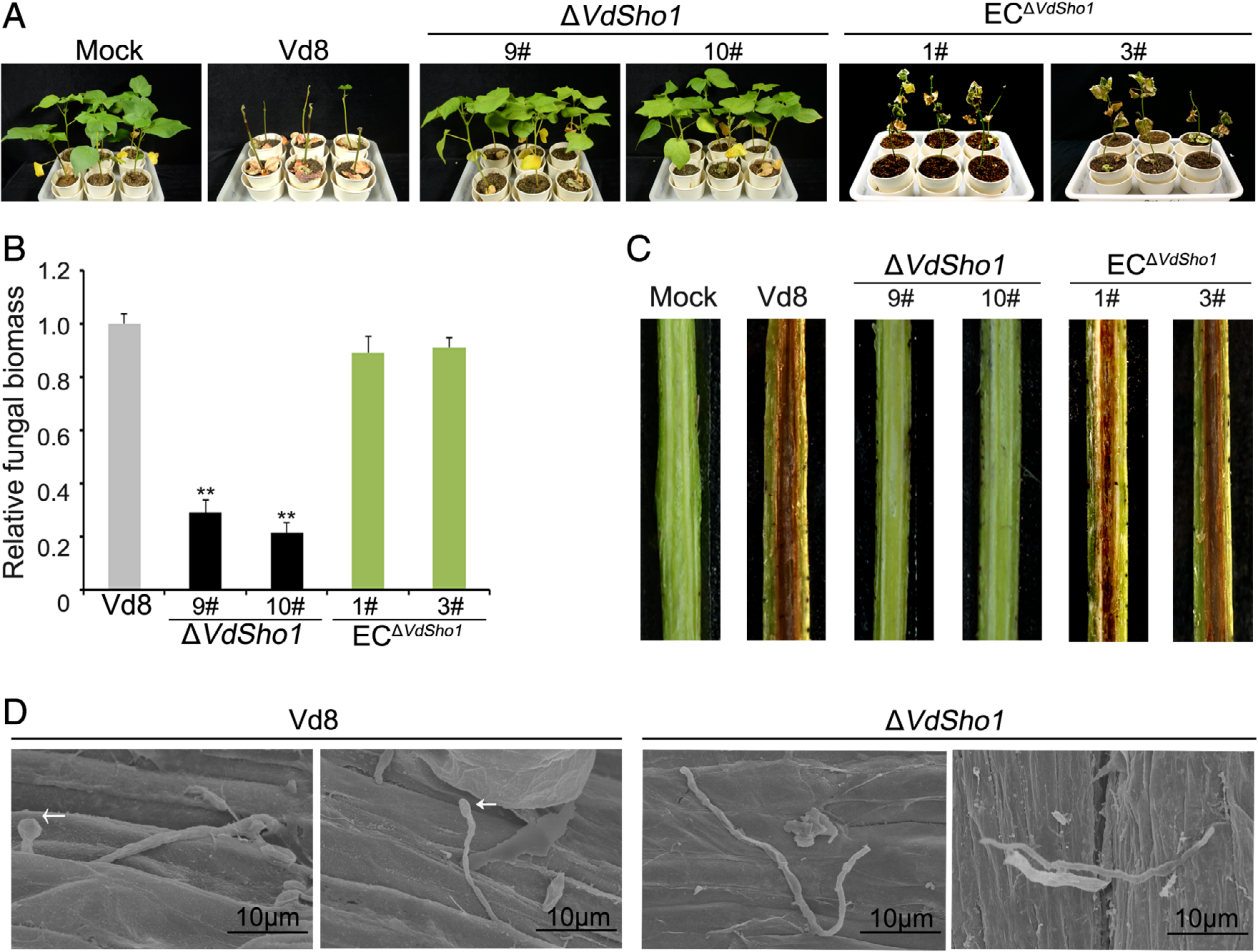
*VdSho1* is required for the full virulence of *Verticillium dahliae* on cotton. (A) Pathogenicity phenotypes of cotton plants inoculated with Δ*Sho1* strains and corresponding complementary transformants. Two‐week‐old seedlings of susceptible cotton (*Gossypium hirsutum* L., *‘*Junmian No. 1’) were inoculated with a 1 × 10^7^ conidia mL^−1^ suspension of wild‐type strain Vd8, the two independent *VdSho1* gene‐deletion strains (Δ*Sho1*‐#9 and Δ*Sho1*‐#10) and the two ectopic transformants (EC^Δ*Sho1*^‐#1 and EC^Δ*Sho1*^‐#3) by a root‐dip method, with three independent replicates and 30 plants per replicate. Cotton seedlings were root‐dipped with sterile water as a control (Mock). The virulence phenotypes were photographed 3 weeks post‐inoculation. (B) Quantitative PCR of fungal biomass (by DNA levels) of the Δ*Sho1* strains and complemented transformants on cotton. Error bars represent the standard deviation, and double asterisks indicate significant differences of Δ*Sho1* strains compared to Vd8 strain at *P* ≤ 0.01 using a Student's *t*‐test. (C) Vascular discoloration of cotton plants inoculated with Vd8, Δ*Sho1* strains and complemented transformants. Uninoculated plants were used as controls (Mock). Vertical sections of cotton hypocotyl were photographed at 3 weeks post‐inoculation with the indicated strains. (D) Investigation of VdSho1 regulating fungal development during cotton root infection by Scanning Electron Microscopy (SEM). Two‐week‐old sterile cotton seedlings were inoculated with the indicated strains (Δ*Sho1* strain and the strain Vd8) for 30 min and then re‐planted into sterile soil. The root tip tissue was harvested for SEM sample processing at 3 days after incubation at 25°C.

The RT‐qPCR analysis revealed that the transcript levels of *VdSho1* during cotton infection were significantly up‐regulated at 24–96 h after inoculation (Fig. S19), providing an initial indication that *VdSho1* is involved in pathogenicity on cotton. To assess the role of *VdSho1* in pathogenicity in *V*. *dahliae*, the pathogenicity of wild‐type strain Vd8, *VdSho1* deletion strains, and the complemented transformants were all assayed by root‐dip inoculation (Liu *et al*., 2013) of a *V*. *dahliae*‐susceptible cotton cultivar. The results revealed that Δ*Sho1* strains resulted in a complete loss of pathogenicity compared with the highly virulent strain Vd8 that caused near death (chlorosis, necrosis and wilting) of inoculated cotton (Fig. 7A), and the fungal biomass in cotton inoculated with the Δ*Sho1* strains was significantly reduced correspondingly (Fig. 7B). The *VdSho1* complemented transformant strains were highly virulent and also showed significantly enhanced fungal biomass relative to the VdSho1 deletion strain (Fig. 7A and B). Analysis of the vascular wilt symptoms further confirmed that Δ*Sho1* strains resulted in a lack of vascular discoloration in cotton, and complementation restored this ability similar to the levels of the wild‐type strain (Fig. 7C). Similarly, following the deletion of *VdSho1* in the hyphal type strain Vd991 (Fig. S6), the strain exhibited significantly reduced pathogenicity and biomass accumulation in cotton (Fig. S20). These results suggested that VdSho1 plays a key role in pathogenicity on cotton. Investigations of the development of strain Vd8 during infection on cotton by electron microscopy showed that the strain developed swollen hyphae, appressoria‐like hyphopodia, and clear penetration pegs to affect plant penetration (Fig. 7B). These data suggest that in *V*. *dahliae*, VdSho1 is required for full virulence of cotton, and the data presented in this study suggest that this occurs through modulation of signalling for membrane penetration via the MAPK pathway that also regulates melanin biosynthesis.

## Discussion

**Figure 8:**
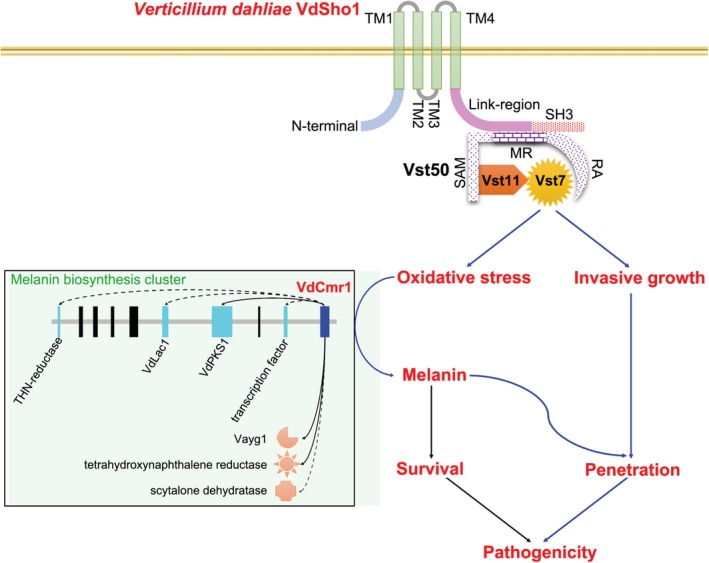
A model of how VdSho1 regulates cotton epidermis penetration and affects melanin biosynthesis in *Verticillium dahliae*. The transmembrane protein, VdSho1, senses membrane permeability to activate the melanin biosynthesis pathway by the MAPK module Vst50‐Vst7‐Vst11 in *V*. *dahliae*, resulting in the accumulation of melanin that promotes survival may counterbalance stressful plant defence responses, but also may have a direct role in penetration of some strains of *V*. *dahliae*. The melanin biosynthesis cluster was drawn according to the reports from Wang *et al*. (2018). The solid arrows represent VdCmr1‐regulated genes important for melanin biosynthesis while dashed arrows represent the melanin biosynthesis‐related genes regulated by VdSho1 in this study.

Sensing the environment and ensuring appropriate cellular responses are crucial challenges confronted by all living organisms, which occur sequentially through recognition, transduction and response pathways (Bahn *et al*., 2007). In this study, the roles of highly conserved sensor protein VdSho1 and its downstream element of MAPK cascades in *V*. *dahliae* were characterized. The results suggest that VdSho1 is required to produce normal hyphopodia, the regulation of melanin biosynthesis, penetration of cellophane membrane, and is necessary for full virulence of *V*. *dahliae* on cotton (Fig. 8A).

Analogous to the signalling function of its orthologs in yeast, the HOG pathway initiated by Sho1 signal transduction pathway generally requires the MAPK cascades of Ste20, Cdc42, Ste11 and Ste50 (Saito and Tatebayashi, 2004; Westfall *et al*., 2004), and ultimately converges at the level of the MAPKK Pbs2 with transduction through the MAPK Hog1 (O'Rourke *et al*., 2002). In *F*. *graminearum*, FgSho1 regulates fungal development and pathogenicity via the MAPK module (Ste50‐Ste11‐Ste7) that Ste50 acts as the scaffolding protein (Gu *et al*., 2015). In this study, we proved again the MAPK module (Ste50‐Ste11‐Ste7) is necessary for the signal transduction from the sensor protein Sho1 in *V*. *dahliae* (Fig. 4B), and further demonstrated that VdSho1 physically interacts with only Vst50 and not with other elements of MAPK cascade (Fig. S10). Unlike the SAM domain of FgSte50 involved in the interaction with FgSho1 (Gu *et al*., 2015), the YTH assays demonstrated that the physical interaction of SH3 domain in VdSho1 with Vst50 through the central conserved region (MR) of unknown function, is independent of SAM and RA domains (Fig. 4B). Sho1 generally regulates the activities of downstream MAPK signalling by influencing the phosphorylation of downstream components in many fungi (Lanver *et al*., 2010; Liu *et al*., 2011; Tatebayashi *et al*., 2015), although the transcriptional level of MAPK components was down‐regulated following the deletion of *VdSho1* in *V*. *dahliae* (Fig. S12). VdSho1 may, therefore, regulate VdSho1‐mediated signalling via MAPKs phosphorylation in addition to affecting the transcript levels of the MAPKs components. Together, these results suggest that fungi employ different regions of scaffolding protein Vst50 to interact with the sensor protein Sho1 and downstream MAPK cascades (Vst11 and Vst7) (Fig. 8), resulting in signal transduction.

In fungi, Sho1 acts as a membrane‐localized sensor that activates the Hog MAPK signalling pathway in response to multiple stress signals, including osmotic stress, hydrogen peroxide adaptation or cell wall integrity stress (Román *et al*., 2005; Gu *et al*., 2015; Tatebayashi *et al*., 2015). When the sensor Sho1 from *M*. *oryzae* was expressed in Δ*Sho1* strains, the penetration ability of mutants was completely restored (Fig. S21). However, several studies of surface sensors have shown that Sho1 displays functional divergence in responding to stress signals in fungi. For instance, Sho1 plays a minor role in the adaptation to osmotic stress in *Candida albicans* but plays a dominant role in its response to oxidative stress and cell wall‐interfering compounds (Román *et al*., 2005), in contrast to Sho1 in *S*. *cerevisiae* that activates the Hog MAPK signalling pathway in response to high osmolarity. Sho1 plays no role in oxidative and osmotic stress in *Alternaria alternata* (Yu *et al*., 2016); FgSho1 is involved in the response to cell wall integrity but not osmotic stress in *F*. *graminearum* (Gu *et al*., 2015). Similarity in *V*. *dahliae*, VdSho1 did not show significant changes in sensitivity to the osmotic stress caused by NaCl or sorbitol, cell wall integrity in response to Congo red or calcofluor white or membrane permeability treatment with nystatin (Figs S13‐S15). Therefore, the function of VdSho1 apparently deviates (independent of HOG pathway) from that of related orthologs in this regard. It was readily apparent in a previous study that the *V*. *dahliae* Hog kinase VdHog1 is required for an appropriate osmotic stress response since the introduction of exogenous NaCl severely restricted growth of the *VdHog1* mutant (Wang *et al*., 2016). However, there are at least two distinct, unrelated and non‐redundant transmembrane proteins, Sln1 and Sho1, regulating adaptation to stress signals (especially osmotic stress) by activating the HOG signalling pathway. In *B*. *cinerea*, BcSHO1 and BcSLN1 redundantly regulate osmotic stress tolerance such that the sensitivity of single deletion mutant to osmotic stress remained unchanged (Ren *et al*., 2019). It is possible that unlike its counterparts in other fungi, VdSho1 does not sense osmotic stress or cell wall integrity but rather this function may be relegated to VdSln1 or by mutual collaboration between VdSho1 and VdSln1 in *V*. *dahliae*, similar to the regulation model in *B*. *cinerea* (Ren *et al*., 2019). The mechanism by which *V*. *dahliae* senses osmotic stress and/or cell wall integrity requires further analysis by double deletion of *VdSho1* and *VdSln1* in a subsequent study.

Previous studies showed that VdSho1 regulates oxidative stress (Qi *et al*., 2016), consistent with the regulation of oxidation‐related gene expression that can modulate melanin biosynthesis (Fig. 2). This is supported by the involvement of Sho1 in the activation of the Ste11‐Ste7‐Kss1 pathway for oxidative stress (Román *et al*., 2005; Ma *et al*., 2008; Xu *et al*., 2016), similar to the involvement of Vst7 in signal transduction for VdSho1 (Fig. 4B; Fig. S10). Furthermore, Sho1 generally is involved in invasive growth via the MAPK component of Ste7 (Bahn *et al*., 2007; Saito, 2010), and its role has been well characterized in the invasive structure development (Raitt *et al*., 2000; Lanver *et al*., 2010; Liu *et al*., 2011; Gu *et al*., 2015). Our studies confirmed VdSho1 regulates the invasive growth via the MAPK component of Vst7, resulting in the defective penetration ability and invasive growth (Figs 1B, C, 5C, and 7D). Therefore, VdSho1 regulates oxidative stress and invasive growth (penetration ability) in *V*. *dahliae*.

VdSho1 and the associated MAPK cascades regulate the melanin biosynthesis in *V*. *dahliae* (Figs 1B, 2, and 5, Fig. S8), and the role of the MAPK pathways in melanin biosynthesis is well characterized (Tzima *et al*., 2010; Tian *et al*., 2014; Tian *et al*., 2016; Wang *et al*., 2016; Yu *et al*., 2019; Zheng *et al*., 2019). Although VdSho1 did not show significant growth changes in response to nystatin, melanin accumulation, however, affected the membrane permeability mediated by nystatin treatment (Fig. S15). VdSho1 sensing the membrane permeability to regulate melanin biosynthesis can also be evidenced by the response of melanin‐related genes to nystatin. Six melanin biosynthesis‐related genes were not sensitive to membrane permeability in the ΔSho1 strains compared to the strain Vd8 and *VdSho1*‐complemented transformants (Fig. 6B). Crosslinking studies indicate that at least one transmembrane domain of Sho1 is required to form planar oligomers of the dimers‐of‐trimers architecture for sensing and signalling transduction by MAPK cascades (Tatebayashi *et al*., 2015). Re‐introduction of the *VdSho1* chimeric genes, which lacked one of four transmembrane domains into the Δ*Sho1* strains failed to restore the ability of the strain to regulate melanin biosynthesis‐related genes under nystatin treatment (Fig. S17), further suggesting that VdSho1 regulates melanin biosynthesis by sensing membrane permeability through the corresponding transmembrane‐associated domains.

Previous studies indicated that melanin deposition is typically associated with microsclerotia formation in *V*. *dahliae* (Duressa *et al*., 2013; Xiong *et al*., 2014). However, melanin production can also be uncoupled from microsclerotia formation. For instance, the *VdPKS1* or *VdCmr1* deletion mutants failed to produce melanin but both could produce albino microsclerotia (Wang *et al*., 2018). Also, based on the growth phenotype on CM agar plates, melanin levels were also slightly reduced in an independent Δ*Sho1* strain of *V*. *dahliae* (Qi *et al*., 2016), which is difficult to reconcile with the findings herein showing a sharp decrease in melanin production in a separate Δ*Sho1* strain. Potentially, some of the differences in melanin production may be strain‐dependent. Moreover, signals from the distinct sensor branches are independently transduced by unique components but converge on common MAPKs (Saito and Tatebayashi, 2004). Thus, potentially other sensors compensate for the lost function of VdSho1, which may result in melanin accumulation following long periods (three weeks) of incubation (Qi *et al*., 2016). The type of medium can also influence whether strains of *V*. *dahliae* produce melanin (Duressa *et al*., 2013). Together, our studies suggested that the sensor protein VdSho1 regulates melanin biosynthesis (oxidative stress) through the membrane permeability and affects the expression of genes associated with melanin biosynthesis in *V*. *dahliae*.

Many recent studies of Sho1 orthologs in fungi have demonstrated that Sho1 plays a critical role in pathogenesis, and this role may overlap with the signal transducing roles of other surface sensors (Msb2, Sln1, etc.) (Lanver *et al*., 2010; Zhang *et al*., 2010; Liu *et al*., 2011; Gu *et al*., 2015; Perez‐Nadales and Di Pietro, 2015; So *et al*., 2018; Ren *et al*., 2019). For instance, FgSho1 and FgSln1 have an additive effect on the pathogenicity of *F*. *graminearum* on wheat (Gu *et al*., 2015). As expected, VdSho1 is required for full virulence of *V*. *dahliae* on cotton and is dependent on the regulation of invasive growth (Fig. 7). For instance, *M*. *oryzae* Sho1 regulates appressorium development that is important for invasive growth (Liu *et al*., 2011).

Melanin is a multifunctional molecule regulating various biological functions in fungi (Wheeler and Bell, 1988; Henson *et al*., 1999). In addition to protecting fungi from extreme environments (Butler and Day, 1998), melanin also plays important roles in their pathogenesis. In *M*. *oryzae*, melanin production governed by the MAP kinase signal transduction pathway is critical to the formation of hyphal cell walls and appressoria (Liu *et al*., 2011). In *V*. *dahliae*, it is clear that melanin protects the fungus from UV irradiation. Therefore, while the deletion of *VdCmr1* or *VdPKS1* abolished melanin production, it had little effect on microsclerotia production. Microsclerotia lacking in melanin are prone to reduced survival, however (Wang *et al*., 2018). Previous studies have provided some clues to melanin regulating the penetration ability of *V*. *dahliae*. For instance, deletion of *VdCrz1*, the transcription factor involved in regulating melanin synthesis, delayed the formation of penetration peg relative to the wild type strain, and significantly reduced the virulence of the mutant strain on cotton (Xiong *et al*., 2015; Zhao *et al*., 2016). In this study, we also found that VdSho1 signalling regulated melanin accumulation that in turn affected morphogenesis in *V*. *dahliae* (Figs 1C and Fig. 7D). In addition to regulating melanin biosynthesis, RNA‐Seq data indicated that multiple other biological functions may compromise aspects of morphogenesis and membrane function following the deletion of *VdSho1* in *V*. *dahliae* (Fig. 2D). Potentially, the regulation of melanin biosynthesis impacts aspects of fungal development and virulence function through VdSho1.

The precise role in the regulation of melanin biosynthesis by VdSho1 remains unclear, and whether or not this function of VdSho1 is directly coupled with virulence remains somewhat controversial. In this study, we demonstrated that *VdSho1* has a role in both the regulation of melanin biosynthesis and in positively affecting the penetration of cellophane membranes. In *M*. *oryzae*, melanin is necessary for the strengthening of the appressorium wall prior to host penetration but this phenomenon has not been so clearly demonstrated in other pathogenic fungi producing appressoria, such as *Alternaria alternata* (Jacobson, 2000). In *V*. *dahliae*, several studies have concluded that melanin accumulation is not required for virulence in *V*. *dahliae* in hosts such as tobacco, tomato and lettuce (Wang *et al*., 2018; Sarmiento‐Villamil *et al*., 2018a; Sarmiento‐Villamil *et al*., 2018b), but another study demonstrated that deletion of *V*. *dahliae* VdPKS1, a key factor for melanin production, resulted in reduced virulence on cotton (Zhang *et al*., 2017). Undoubtedly, gene deletion studies of various MAPK cascade components suggest a strong correlation between melanin biosynthesis and virulence in *V*. *dahliae* (Tzima *et al*., 2010; Tian *et al*., 2014; Tian *et al*., 2016; Wang *et al*., 2016; Yu *et al*., 2019; Zheng *et al*., 2019). Thus, the degree of influence of melanin may be contingent on the strain, and the host type challenged. Regardless of whether or not DHN melanin production is a prerequisite for virulence in *V*. *dahliae*, melanin is clearly required for increased survival in response to stress response (Wang *et al*., 2018; Fang *et al*., 2019). However, some stress responses did not change in the Δ*Sho1* strain in *V*. *dahliae* as compared to the wild‐type strain (Fig. S22). Interestingly, the *ΔSho1* strain lost its ability to penetrate the cellophane membrane. The cellophane membrane penetration or lack thereof was also correlated with increased or reduced melanin levels as in the typical hyphal strain (Fig. 3D and E; Fig. S5, S6) and melanin inhibitors such as carpropamid and tricyclazole block cellophane membrane penetration by *V*. *dahliae* (Fig. 3A–C; Fig. S4). However, it is possible that VdSho1 regulates other secondary metabolite clusters (directly or indirectly) in addition to melanin biosynthesis as many other oxidation‐related genes (more than 70 genes) are also regulated by VdSho1 (Figs 2D and E, Table S1). In *F*. *graminearum*, FgSho1 can regulate mycotoxin production that in turn can also affect virulence on wheat (Gu *et al*., 2015). Together, the studies incorporating the inhibitors carpropamid and tricyclazole suggest that melanin regulation by VdSho1 affects pathogenicity in correlation with invasive growth (formation of hyphopodia/penetration) during infection of cotton (Fig. 8A) and that this may be *V*. *dahliae* strain‐host dependent, though a lack of melanin may also influence survival and stress responses even on cellophane membranes because it is well established that melanin plays roles in protection against various cellular stresses (Butler and Day, 1998; Wang *et al*., 2018; Fang *et al*., 2019).

In summary, cotton infection and host colonization in *V*. *dahliae* is initiated and sustained by functional VdSho1‐mediated signal transduction via the Vst50‐Vst7‐Vst11 module. VdSho1‐mediated signalling pathways regulate penetration and melanin biosynthesis, resulting in *V*. *dahliae* colonization and virulence on cotton. Potentially, reduced melanin biosynthesis, such as in the Δ*Sho1* strain described in this study, affects the proper stress responses in the pathogen during host infection and colonization, and even *in vitro* when breaching cellophane membranes. Eventually, novel methods of disease control may be achieved by exploiting components of signal transduction pathways that affect infection and interfere with pathogen survival in the soil and *in planta*, in response to various stresses.

## Experimental Procedures

### *Fungal culture conditions and transformation*

The wild‐type strains Vd8 and Vd991 (Zhou *et al*., 2013; Chen *et al*., 2018) used in this article were collected from infected cotton plants as also the strains 1‐2, 16‐1, 19‐3, 08026. Cultures were maintained on potato dextrose agar medium or in liquid Czapek Dox medium for 5 days at 25°C. Gene deletion strains were produced using ATMT method as described previously (Liu *et al*., 2013). To generate *VdSho1* deletion constructs, the flanking sequences of *VdSho1* were amplified from either Vd8 or Vd991 genomic DNA as appropriate and integrated with the hygromycin resistance cassette using fusion PCR following the procedure of Liu *et al*. (2013). The amplified products were cloned into the binary vector pDht2 (Zhou *et al*., 2013). Positive gene deletion strains were verified by DNA blotting analysis using a DIG High Prime DNA Labelling and Detection Starter Kit II (Roche, Penzberg, Germany). Hygromycin gene‐specific DNA probes were amplified using the corresponding primers (Table S2). To generate complementation transformants, the genomic region encoding VdSho1 was amplified from the genomic DNA of each of the strains Vd8 and Vd991 and cloned into the binary vector pCOM that carries geneticin resistance (Zhou *et al*., 2013), and reintroduced to the *∆Sho1* strains. For the analysis of the genetic relationship between *Vst50* and *VdSho1*, the *Vst50* complementation construct pCOM vector containing the genomic coding region of *Vst50* was transformed into the *∆Sho1* strain while the *VdSho1* complementation construct was transformed into the *∆Vst50* strain. For ectopic expression of *MoSho1*, a full‐length sequence of *MoSho1* was cloned from the *M*. *oryzae* genomic DNA and cloned into the binary vector pCOM containing *VdSho1* promoter and terminator. For functional domain analysis of *VdSho1*, the cDNA sequence of *VdSho1*, whereby domain‐specific mutations were introduced, were cloned into the binary vector pCOM containing *VdSho1* promoter and terminator and reintroduced to the *∆Sho1* strains. For functional analysis of Vst50 domains, the cDNA sequence of *Vst50* containing the desired mutations was cloned into the binary vector pCOM containing the *Vst50* promoter and terminator and reintroduced to the *∆Vst50* strain. Complementary transformants were selected on PDA (potato, 200 g L^−1^, glucose, 20 g L^−1^, agar, 15 g L^−1^) containing antibiotics (60 μg ml^−1^ hygromycin, 50 μg ml^−1^ geneticin). Gene deletion mutants and ectopic transformants were verified by PCR with the corresponding primers (Table S2).

### *Pathogenicity assays*

Two‐week‐old cotton seedlings were inoculated with an inoculum suspension of 5 × 10^6^ conidia ml^−1^ of the corresponding strains by the root‐dip method (Liu *et al*., 2013). Disease index recordings were initiated at 9 days post‐inoculation until the plants inoculated with wild‐type strains exhibited total wilting or were dead. Vascular discoloration of infected cotton was observed in longitudinal sections of the shoots 3 weeks after inoculation. For fungal biomass quantification, the roots of nine plants were harvested at 21 days after inoculation for genomic DNA extraction. Quantitative PCR was performed following the procedure of Santhanam and Thomma (2013) for the quantification of fungal biomass. Fungal colonization levels were derived from DNA amplification of the *Verticillium* elongation factor 1‐a (EF‐1a), which were normalized to the expression of the cotton 18S gene. The growth of all test strains during cotton infection was investigated by scanning electron microscopy, as described previously (Jin *et al*., 2011).

### *Penetration assay*

Equal amounts of conidia collected from the corresponding *V*. *dahliae* strains were grown on the top of cellophane membranes (Solarbio, Beijing, China) that were placed on MM for a desired number of days at 25°C. The cellophane membranes were removed from the plates and the plates were further incubated for 3 additional days to determine if *V*. *dahliae* growth following cellophane membrane penetration. The penetration progress of the individual strains on the cellophane membrane was investigated by transmission electron microscopy as described previously (Li *et al*., 2006).

### *Yeast two‐hybrid*

YTH analysis was performed as described previously (Tang *et al*., 2012). Interactions between test groups were quantified according to the method described in Yeast Protocols Handbook (Clontech, Mountain View, CA, USA). The cDNA of bait and prey genes was inserted into yeast GAL4‐binding domain containing vector pGBKT7 and GAL4 activation domain containing vector pGADT7, respectively. The coding sequences of the tested genes to be used as bait or prey were amplified from the cDNA derived from *V*. *dahliae* strain Vd8 with the appropriate primer pairs (Table S2). Two hybrid interaction‐positive strains were verified by growth on SD (‐Leu/‐Trp/‐His/‐Ade). The interaction of murine p53 (p53) and SV40 large T‐antigen (T) was used as a positive control for the system, and human lamin C (lam) as a negative control.

### *Gene Expression Analysis*

For *VdSho1* gene expression during cotton infection, 3‐week‐old cotton seedlings were inoculated with 5 ml of 1 × 10^7^ conidia ml^−1^ of a *V*. *dahliae* conidial suspension from the wild‐type strain Vd8 by root‐dip method (Hu *et al*., 2015). The roots were harvested at 24, 48, 72, 96 and 120 h post‐inoculation for RNA extraction. The wild‐type Vd8 strain cultured on PDA medium was used as an *in vitro* control for comparisons. Total RNA was extracted using the AxyPrep Multisource Total RNA Miniprep Kit (Axygen), and first‐strand cDNA was synthesized using reverse transcriptase (Invitrogen). Quantitative real‐time PCR was carried out using the SYBR Premix ExTaq Kit (Takara) following the manufacturer's instructions. Gene expression levels of *VdSho1* were normalized to the expression of the *Verticillium* EF‐1a.

For expression of melanin synthesis‐related genes during the early interactions of *V*. *dahliae* on the cellophane membrane and during membrane penetration, strains were grown on the top of a cellophane membrane that was placed on MM for 3 days at 25°C. The samples were harvested for analysis of transcript levels of melanin biosynthesis‐related genes, and the housekeeping gene *β‐tubulin* (*VDAG_10074*) was used as an endogenous control. Primer sequences used are listed in Table S2.

### *RNA‐seq and gene expression profile analysis*

High conidial suspension concentrations (1 × 10^8^ conidial ml^−1^) of wild‐type Vd8, ∆*Sho1*(Δ*Sho1*,10#), and the ∆*Vst50* strain were incubated on the cellophane membranes placed on MM at 25°C for 3 days. Total RNA was extracted for RNA‐sequencing. The strain Vd8, ∆*Sho1* strain and ∆*Vst50* strain grown on the cellophane membranes included Vd8 (mem), *ΔSho1* (mem) and *ΔVst50* (mem) respectively. The Vd8 strain cultured on MM was set as a control. Transcriptome profile analysis was done as described previously (Zhang *et al*., 2017).

### *Strain stress response assay*

For osmotic stress assays, complete medium (CM) plates were supplemented with 1 M NaCl or 1.5 M sorbitol respectively. CM plates without such supplements were set as controls. Drops of spore suspensions diluted in a water gradient (10^6^, 10^5^, 10^4^, 10^3^ conidia ml^−1^) were cultured on the CM plates with or without the supplements and incubated at 25°C. Colony diameters were measured after 5 days of incubation. The Kruskal–Wallis analysis of variance and the Mann–Whitney tests were used to assess statistically significant differences among strains at *P* ≤ 0.05.

For cell wall stress assays, CM plates were supplemented with 200 mg mL^−1^ Congo Red (Sigma‐Aldrich) or 150 mg ml^−1^ Calcofluor white (Sigma‐Aldrich) respectively. CM plates without supplements were set as controls. A series of concentrations of spores were cultured on CM plates with or without supplements and incubated at 25°C. Colony diameters on all plates were measured after 5 days of incubation. The Kruskal–Wallis analysis of variance and the Mann–Whitney tests (Gui *et al*., 2017) were used to assess statistically significant differences among strains at *P* ≤ 0.05.

### *Microsclerotia assay*

The microsclerotia production was assayed as previously described (Wang *et al*., 2016). In detail, the conidial suspension of 10 μl each strain was sprayed onto the cellophane membrane, which had been overlaid on solid Basal modified medium (glucose, 5.0 g L^−1^; NaNO_3_, 0.2 g L^−1^; KCl, 0.52 g L^−1^; MgSO_4_•7H_2_O, 0.52 g L^−1^; K_2_HPO_4_, 1.52 g L^−1^; vitamin B1, 3.0 μM; vitamin H, 0.1 μM; agar, 15 g L^−1^) (Bai *et al*., 2011). Developmental stages of microsclerotia were observed under light microscopy (DM2500, Leica) at 1‐week post‐incubation.

### *Bioinformatics analysis*

Full‐length protein sequences of the VdSho1 homolog from *V*. *dahliae* and from other fungi were downloaded from NCBI database following BLAST searches with VdSho1 as the query. Clustal X1.83 was used for multiple sequence alignments and DNAMAN was used for the alignment picture output. Protein domain predictions were made using SMART (http://smart.embl-heidelberg.de/).

## Additional reagents

The following reagents were used in the penetration assays. Melanin synthesis inhibitors: tricyclazole (Macklin) and carpropamid (Aladdin), polyene antibiotic nystatin (TargetMol).

## Author's Contributions

X.‐F.D., J.‐Y.C. and K.V.S. conceived this research. J.‐Y.C., L.Z., and J.‐J.L. designed and directed the study. J.‐J.L. and L.Z. performed the functional characterization of VdSho1. X.‐P.H. cooperated with the functional characterization of VdPKS1. J.‐Y.C. performed the bioinformatics analyses. J.‐Y.C. and J.‐J.L. processed the figures and data. D.‐D.Z., J.‐J.L., J.S., B.‐L.W., and D.W. participated in research and discussion. J.‐Y.C., L.Z., J.‐J.L., and S.J.K. wrote the first draft of the manuscript. K.V.S. and S.J.K. edited the manuscript.

## Supporting information


**Fig. S1. Comparison of protein sequence divergence between VdSho1 in *Verticillium dahliae* strain Vd8 and homologous gene products from *V*. *longisporum*, *S*. *cerevisiae*, *M*. *oryzae*, *F*. *oxysporum* and *F*. *graminearum***. The full‐length protein sequences of VdSho1 and its corresponding orthologs in representative fungi were downloaded from the NCBI database. Clustal X 1.83 was used for multiple sequence alignments and DNAMAN was used for the alignment graphical output. Conserved sites are listed under the sequence alignments. The secondary structure assignments of the VdSho1 protein are depicted at the bottom of the alignment.
**Fig. S2. VdSho1 deletion and complementation in *Verticillium dahliae*** (**a**) Schematic map of the homologous recombination event involving Δ*Sho1* mutants; (**b**) Southern blotting assay to analyse T‐DNA insertion copies in *VdSho1* gene deletion strains; (**c**) Identification of complemented transformants using hygromycin resistance gene internal primers; (**d**) Identification of complemented transformants using *VdSho1* gene amplification primers; (**e**) Identification of complemented transformants using geneticin resistance gene internal primers.
**Fig. S3. Penetration of cellophane membrane by *Verticillium dahliae***. The strain Vd8 was grown on cellophane membranes overlaid on minimal medium (MM) and incubated for two or three days at 25°C. The cellophane membranes were removed from the plates and examined for penetration by transmission electron microscopy. Blue arrow represents a conidium that has germinated and penetrated the cellophane membrane.
**Fig. S4. Melanin accumulation in *Verticillium dahliae* following treatment with carpropamid**. (**a**) Inhibition of melanin biosynthesis by carpropamid is correlated with reduced ability of *V*. *dahliae* to penetrate cellophane membranes. The wild‐type strain Vd8 was grown on cellophane membranes overlaid on minimal medium (MM) and the melanin biosynthesis inhibitor of tricyclazole (0, 0.5 and 1.5μg mL^‐1^) and incubated for three days at 25°C (set as “Before” status). The cellophane membranes were removed from the plates and incubated for an additional three days to determine penetration as indicated by mycelial growth on the medium (set as “After” status). The strain grown on the purified MM medium was set as control (Mock). (**b**) Gene expression analysis of melanin biosynthesis‐related genes in response to carpropamid. The Vd8 strain was grown on cellophane membranes overlaid on MM medium with the carpropamid, and the tissue was harvested after three days incubation at 25°C. RT‐qPCR was performed to determine the expression levels of melanin biosynthesis‐related genes on the MM medium with carpropamid treatment, relative to growth on the MM only control (Mock).
**Fig. S5. Cellophane membrane penetration by four *Verticillium dahliae* strains**. (**a**,**c**,**e**,**g**) Hyphal type strains 1‐2,16‐1,19‐3 and 08026 were grown on cellophane membranes overlaid on MM medium for three or four days at 25°C. Cellophane membranes were removed from the plates and incubated for another three days at 25°C. Melanin accumulation was determined by examining the darkened underside of plates. (**b**,**d**,**f**,**h**) Expression levels of melanin biosynthesis‐related genes during cellophane membrane penetration in the hyphal type strains 1‐2, 16‐1,19‐3 and 08026. RT‐qPCR was performed in two hyphal type strains three and four days after incubation on the top of cellophane membranes at 25°C. Error bars represent the standard deviation associated with three replicate experiments, and **indicates statistical significance (*P* ≤ 0.01) determined by an unpaired Student's *t*‐test.
**Fig. S6. Melanin accumulation correlates with cellophane membrane penetration in *Verticillium dahliae* hyphal type strain Vd991**. (**a**) Cellophane membrane penetration and examination of melanin accumulation in the hyphal type strain Vd991. The hyphal type strain Vd991 was grown on the top of cellophane membranes for periods three, four or seven days at 25°C. The cellophane membranes were removed from the plates and incubated an additional three days. Melanin accumulation was determined by the darkened underside of plates. (**b**) Expression of melanin biosynthesis‐related genes during cellophane membrane penetration in the hyphal type strain Vd991. RT‐qPCR was performed to determine the expression levels of melanin biosynthesis‐related genes in the hyphal type strain Vd991 following three, four, or seven days of incubation on cellophane membranes at 25°C. Vd8 strain was grown for three days as controls.
**Fig. S7. The melanin biosynthesis mediated by VdPKS1 is correlated with cellophane membrane penetration in *Verticillium dahliae***. Strains including the *VdSho1* deletion strain, the *VdPKS1* gene‐deletion strain, the complementary transformant (EC^Δ*PKS1*^), and wild‐type strain Vd8, were grown on cellophane membranes overlaid onto minimal medium (MM) for three days at 25°C (“Before” status). The cellophane membranes were removed from the plates and the plates were incubated for an additional three days to determine the cellophane membrane penetration by the presence or absence of mycelial growth on the medium (“After” status).
**Fig. S8. The *Verticillium dahliae* VdSho1 modulates cellophane membrane penetration through the adaptor Vst50 and regulates melanin‐associated gene expression**. (**a**) Identification of the genes regulated by Vst50 during cellophane membrane penetration. The flowchart of determining the genes regulated by Vst50 is shown in Fig. 3a. (**b**) Gene regulation by VdSho1 and Vst50. Vst50/VdSho1 (red box‐whisker plot) represents the ratio of the fold‐change regulated by VdSho1 [*ΔSho1* (mem) *vs* (Vd8(mem)]; Vst50 [*ΔSte50* (mem) *Vs* (Vd8(mem)]. Vst50/Vd8 (green box‐whisker plot) represents the ratio of the fold‐change regulated by Vst50 [*ΔSte50* (mem) *Vs* (Vd8(mem)]; [Vd8(mem) *Vs* Vd8(MM)] during the cellophane membrane penetration. In each box plot, the central point represents the median, the rectangle provides the interval between the 25% and 75% percentiles. (**c**) Melanin biosynthesis‐related genes regulated by VdSho1 and Vst50. The green colour represents the genes down‐regulated after deletion of Vst50. (**d**) Melanin accumulation regulated by Vst50. Strains, including the *Vst50* gene‐deletion (Δ*Vst50*), the complemented transformant (EC^Δ*Vst50*^) and the wild‐type strain Vd8 were grown on top of cellophane membranes on MM medium and the phenotypes were photographed under microscopy three days after incubation at 25°C. (**e**) RT‐qPCR analysis of the expression of melanin biosynthesis‐related genes regulated by Vst50 during cellophane membrane penetration. The samples were harvested from the wild‐type strain Vd8, *Vst50* deletion strains (Δ*Vst50*), and ectopic transformants (EC^Δ*Vst50*^) that were grown on top of cellophane membranes on MM medium. *β‐tubulin* (*VDAG_10074*) was used as an endogenous control of gene expression. Error bars represent the standard deviation of three replicate experiments, and **indicate statistical significance (*P* ≤ 0.01) determined by an unpaired Student's *t*‐test. (**f**) *Vst50* overexpression rescues the ability of *VdSho1* deletion strain to penetrate cellophane membrane. The strains indicated in **Fig. 3c** were grown on the cellophane membranes overlaid on minimal medium (MM) for three days at 25°C at which time the samples were harvested for the transcript level analysis of melanin biosynthesis‐related genes. *β‐tubulin* (*VDAG_10074*) was used as an endogenous control. Error bars represent the standard deviation of three replicate experiments, and **indicates statistical significance (*P* ≤ 0.01) determined by an unpaired Student's *t*‐test.
**Fig. S9. Reverse transcription quantitative PCR (RT‐qPCR) analyses of the expression of *Vst50* and *VdSho1* in ectopic transformants**. (**a**) Analysis of *Vst50* expression in Vd8, Δ*Sho1* and Vst50/Δ*Sho1* strains. The indicated strains were cultured on the top of cellophane membranes on minimal medium (MM) for three days at 25°C. The samples were harvested for the transcript levels for RT‐qPCR analyses of melanin biosynthesis related genes. *β‐tubulin* (*VDAG_10074*) was used as an endogenous control for gene expression. Error bars represent the standard deviation of three replicate experiments, and **indicates statistical significance (*P* ≤ 0.01) determined by an unpaired Student's *t*‐test. (**b**) RT‐qPCR analysis of *VdSho1* expression in Vd8, Δ*Vst50*, *VdSho1*/Δ*Vst50* and *Vst50* ^*ΔMR*^ */ΔVst50* strains.
**Fig. S10. Analysis of protein‐protein interaction by yeast two hybrid (YTH) assays**. (**a**) Identification of the physical interactions between VdSho1 and downstream signalling components by YTH assay. The region encoding the intracellular portion of VdSho1 was incorporated into construct pGBKT7 as bait while the cDNAs encoding downstream regulatory factors of *Vst7*, *Vst11*, *Vst20*, *Vst50*, *VdVMK1*, and *VdCdc42* were used as prey to construct into pGADT7 vector. Ten‐fold serially diluted yeast strains were simultaneously cultured on SD (‐Leu/‐Trp/‐His/‐Ade) plates, where only protein interaction‐positive strains should grow. (**b**) Identification of the physical interactions between Vst7 and Vst11 by YTH assays. Vst7 was used as bait to construct pGBKT7 vector and Vst11 was set as prey to construct into pGADT7 vector. Ten‐fold serially diluted yeast strains were simultaneously cultured on SD (‐Leu/‐Trp/‐His/‐Ade) plates, where only bait‐prey interaction‐positive strains grow. In each of these experiments, the interaction of murine p53 (p53) and SV40 large T‐antigen (T) was used as a positive control for the system, and human lamin C (lam) was used as a negative control.
**Fig. S11. RA and SAM domains in Vst50 of *Verticillium dahliae* are essential for cellophane membrane penetration and melanin biosynthesis**. (a) Identification of the cellophane membrane penetration ability of *Vst50* using full length *Vst50*, and RA, SAM domain deletion mutants. All of the indicated strains were grown on cellophane membranes overlaid on minimal medium (MM) for three days at 25°C (set as “Before” status). The cellophane membranes were removed from the plates after an additional three days of incubation to determine whether cellophane membrane penetration had occurred, by examining the presence or absence of mycelial growth on the medium (set as “After” status). (**b**) Reverse transcription quantitative PCR (RT‐qPCR) analysis of the expression of melanin biosynthesis‐related genes in the corresponding strains. The strains were grown on cellophane membranes overlaid on minimal medium (MM) for three days at 25°C. Following this three‐day incubation, the samples were harvested for analysis of melanin biosynthesis‐related gene expression by RT‐qPCR. The housekeeping gene *β‐tubulin* (*VDAG_10074*) was used as an endogenous control for gene expression. Error bars represent the standard deviation of three replicate experiments; **indicates statistical significance (*P* ≤ 0.01) determined by an unpaired Student's *t*‐test.
**Fig. S12. VdSho1 regulates the expression of MAPK cascade genes in *Verticillium dahliae***. Reverse transcription quantitative PCR (RT‐qPCR) analysis the expression of MAPK cascade related genes in the wild‐type strain Vd8 and Δ*Sho1* strains. Strains were cultured in liquid Czapek Dox medium for 5 d at 25°C and mycelia were harvested for RT‐qPCR analyses of MAPK cascade related gene expression. The housekeeping gene *β‐tubulin* (*VDAG_10074*) was used as an endogenous control. Error bars represent the standard deviation of three replicate experiments and **indicates statistical significance (*P* ≤ 0.01) determined by an unpaired Student's *t*‐test.
**Fig. S13. Analysis of a role of VdSho1 in osmotic and salt stress defence response in *Verticillium dahliae***. **(a)** Involvement of VdSho1 in the response to osmotic and salt stress. A drop of 10^6^ conidia mL^‐1^ suspensions of the wild‐type Vd8 and Δ*Sho1* strains was placed in the centre of complete medium (CM), CM supplemented with 1 M NaCl, or 1.5 M sorbitol for five days at 25°C. Bar = 1 cm. **(b)** Colony diameters were measured after 4 days incubation. Error bars represent standard deviations of five independent plates. The same letter on mean values indicates that they were not significantly different according to Mann–Whitney test (*P* ≤ 0.05).
**Fig. S14. VdSho1 is not required for cell wall stress response in *Verticillium dahliae***. **(a)** Analysis of VdSho1 in response to cell wall perturbations. Conidia suspension of wild‐type strain Vd8 and Δ*Sho1* strains were grown on complete medium (CM), CM supplemented with 200 μg mL^‐1^ Congo red, or 150 μg mL^‐1^ calcofluor white (CFW) for five days at 25°C. Bar = 1 cm. **(b)** Colony diameters following growth of the fungus from a single drop of 10^6^ conidia mL^‐1^ placed in the centre of the plates were measured after 4 days incubation. Error bars represent standard deviations of five independent plates. Mean values followed by the same letters indicate that they were not significantly different according to Mann–Whitney test (*P* ≤ 0.05).
**Fig. S15. The membrane permeability sensed by VdSho1 is required for melanin accumulation in *Verticillium dahliae***. **(a)** Analysis of VdSho1 in response to membrane permeability altered by nystatin. A single drop of 10^6^ spores mL^‐1^ conidial suspension of wild‐type strain Vd8, ΔSho1, and the complemented mutant strain were placed on complete medium (CM), or CM supplemented with 0.5 and 1.0 μg mL^‐1^ nystatin for five days at 25°C. **(b)** Colony diameters were measured after four days of incubation. Error bars represent standard deviations of five independent plates. The same letter associated with the mean values indicates lack of statistical significance according to Mann–Whitney test (*P* ≤ 0.05).
**Fig. S16. VdSho1 positively regulates the expression of melanin biosynthesis‐related genes**. (**a**) Reverse transcription quantitative PCR (RT‐qPCR) analysis of the expression level of *VdSho1* in *VdSho1* complemented transformants. The strains were grown on the cellophane membranes overlaid on the minimal medium (MM) for three days at 25°C and samples were harvested for RT‐qPCR analyses immediately after. *β‐tubulin* (*VDAG_10074*) was used as an endogenous control for gene expression analysis. Error bars represent the standard deviation of three replicate experiments. * indicates statistical significance (*P* ≤ 0.05) determined by an unpaired Student's *t*‐test and **indicates statistical significance (*P* ≤ 0.01) determined by an unpaired Student's *t*‐test. (**b**) RT‐qPCR analysis of the expression level of melanin biosynthesis‐related genes in two independent *VdSho1* complemented transformants.
**Fig. S17. The transmembrane domains of VdSho1 in *Verticillium dahliae* are essential for melanin production**. Reverse transcription quantitative PCR (RT‐qPCR) analysis of the expression of melanin biosynthesis‐related genes in the complemented transformants (transmembrane domain deletion of VdSho1). The strains were grown on cellophane membranes overlaid on minimal medium (MM) for three days at 25°C. Samples were harvested for RT‐qPCR analysis of melanin biosynthesis‐related genes immediately thereafter. *β‐tubulin* (*VDAG_10074*) was used as an endogenous control. Error bars represent the standard deviation of three replicate experiments and **indicates statistical significance (*P* ≤ 0.01) based on unpaired Student's *t*‐test.
**Fig. S18. Gene expression analysis of melanin biosynthesis‐related genes in the complemented transformants of *Verticillium dahliae* (introduced transmembrane domain deletion of VdSho1) response to nystatin**. (**a**) The strains were grown on cellophane membranes overlaid on minimal medium (MM) with nystatin for three days at 25°C and samples were harvested to ascertain transcripts levels of melanin biosynthesis‐related genes. The *V*. *dahliae β‐tubulin* (*VDAG_10074*) was used as an endogenous control for gene expression analysis. Bars in blue, red and green represent respective strains grown with 0, 0.5 and 1.0 μg mL^‐1^ nystatin respectively. Error bars represent the standard deviation of three replicate experiments; red star, red dot, and circle with cross line indicates statistical significance at *P* ≤ 0.001, *P* ≤ 0.01, and *P* ≤ 0.05 based on unpaired Student's *t*‐tests respectively.
**Fig. S19. Expression analysis of *VdSho1* during infection of *Verticillium dahliae* on cotton**. Three‐week‐old cotton plants (*G*. *hirsutum* cv. Junmian No.1) were inoculated with the *V*. *dahliae* wild‐type strain Vd8 by root‐dip method and harvested at regular intervals from 0 to 120 h post inoculation. Cotton roots were harvested for the transcripts level analysis of *VdSho1* by reverse transcription quantitative PCR. *V*. *dahliae* translation *β‐tubulin* (*VDAG_10074*) was used as an endogenous control for gene expression analysis. Error bars represent the standard deviation of three replicate experiments and **indicates statistical significance (*P* ≤ 0.01) based on unpaired Student's *t*‐test.
**Fig. S20. VdSho1 is required for the full virulence of *Verticillium dahliae* strain Vd991 on cotton**. (**a**) Pathogenicity phenotypes of *VdSho1* gene deletion strain and the corresponding complemented transformant inoculated on cotton. Two‐week‐old seedlings of susceptible cotton (*Gossypium hirsutum* cv. Junmian No. 1) were inoculated with a 1×10^7^ conidia mL^‐1^ suspension of wild‐type strain Vd991, the *VdSho1* gene‐deletion strain (Δ*Sho1*) and the ectopic transformant (EC^ΔSho1^) by root‐dip method, with three independent replicates and 30 plants for each strain within each replicate. Cotton seedlings root‐dipped with the sterile water were used as uninoculated control. The virulence phenotypes were photographed three weeks post inoculation. (**b**) Quantitative PCR quantification of fungal biomass of the *VdSho1* gene deletion strain and complemented transformant in cotton. Error bars represent the standard deviation and double asterisks (**) indicate significant differences of *VdSho1* gene‐deletion strains compared with Vd991 strain at *P*≤ 0.01 using Student's *t*‐test. (**c**) Vascular discoloration of cotton plants inoculated with wide‐type strain Vd8, Δ*Sho1* strain and complementary transformant. Vertical sections of cotton hypocotyl were photographed at three weeks post inoculation with the indicated strains.
**Fig. S21. Functional homology analysis of MoSho1 and VdSho1**. **(a)** Cellophane membrane penetration ability of *MoSho1*/ΔSho1 transformants. All strains were grown on cellophane membranes overlaid on minimal medium (MM) for three days at 25°C (set as “Before” status). The cellophane membranes were removed from the plates and incubated for an additional three days to determine the cellophane membrane penetration by mycelial growth on medium (“After” status). (**b**) Reverse transcription quantitative PCR (RT‐qPCR) analysis of the expression of melanin biosynthesis‐related genes in *MoSho1*/Δ*Sho1* transformants. The strains were grown on the top of cellophane membranes on minimal medium (MM) for three days at 25°C and the samples were harvested for RT‐qPCR analysis of melanin biosynthesis‐related genes. *β‐tubulin* (*VDAG_10074*) was used as an endogenous control for gene expression analysis. Error bars represent the standard deviation of three replicate experiments and **indicates statistical significance (*P* ≤ 0.01) based on an unpaired Student's *t*‐test.
**Fig. S22. Assays of the role of VdSho1 in microsclerotium production in *Verticillium dahliae***. Microsclerotia formation in wild‐type strain Vd8 and *VdSho1* deletion strains. A 150 μL 10^6^ conidia mL^‐1^ spore suspension of, each strain was spread onto BMM and incubated at 20°C in the dark. Microscopy images were taken of each genotype after one week of incubation.

**Table S1 Analysis the transcriptome mediated by VdSho1 and Vst50 during cellophane membranes penetration in *Verticillium dahliae***
(Note of regulation patters, T1: Up‐regulation to Down‐regulation; T2: Down‐regulation to Up‐regulation; T3: Up‐regulation to Up‐regulation; T4: Down‐regulation to Down‐regulation)

**Table S2** Primers used in this study
