# Supplementary material for: The Verticillium dahliae Sho1‐MAPK pathway regulates melanin biosynthesis and is required for cotton infection
Source: Environ Microbiol. 2019 Nov 24;21(12):4852–74. doi: 10.1111/1462-2920.14846 (PMC6916341; doi:10.1111/1462-2920.14846)
Supplement: Supplementary file 1 — Fig. S1. Comparison of protein sequence divergence between VdSho1 in Verticillium dahliae strain Vd8 and homologous gene products from V. longisporum, S. cerevisiae, M. oryzae, F. oxysporum and F. graminearum. The full‐length protein sequences of VdSho1 and its corresponding orthologs in representative fungi were downloaded from the NCBI database. Clustal X 1.83 was used for multiple sequence alignments and DNAMAN was used for the alignment graphical output. Conserved sites are listed under the sequence alignments. The secondary structure assignments of the VdSho1 protein are depicted at the bottom of the alignment. Fig. S2. VdSho1 deletion and complementation in Verticillium dahliae (a) Schematic map of the homologous recombination event involving ΔSho1 mutants; (b) Southern blotting assay to analyse T‐DNA insertion copies in VdSho1 gene deletion strains; (c) Identification of complemented transformants using hygromycin resistance gene internal primers; (d) Identification of complemented transformants using VdSho1 gene amplification primers; (e) Identification of complemented transformants using geneticin resistance gene internal primers. Fig. S3. Penetration of cellophane membrane by Verticillium dahliae. The strain Vd8 was grown on cellophane membranes overlaid on minimal medium (MM) and incubated for two or three days at 25°C. The cellophane membranes were removed from the plates and examined for penetration by transmission electron microscopy. Blue arrow represents a conidium that has germinated and penetrated the cellophane membrane. Fig. S4. Melanin accumulation in Verticillium dahliae following treatment with carpropamid. (a) Inhibition of melanin biosynthesis by carpropamid is correlated with reduced ability of V. dahliae to penetrate cellophane membranes. The wild‐type strain Vd8 was grown on cellophane membranes overlaid on minimal medium (MM) and the melanin biosynthesis inhibitor of tricyclazole (0, 0.5 and 1.5μg mL‐1) and incubated for three days at 25 [file EMI-21-4852-s001.docx]

**Supplementary Figures**

**Figure S1**

**
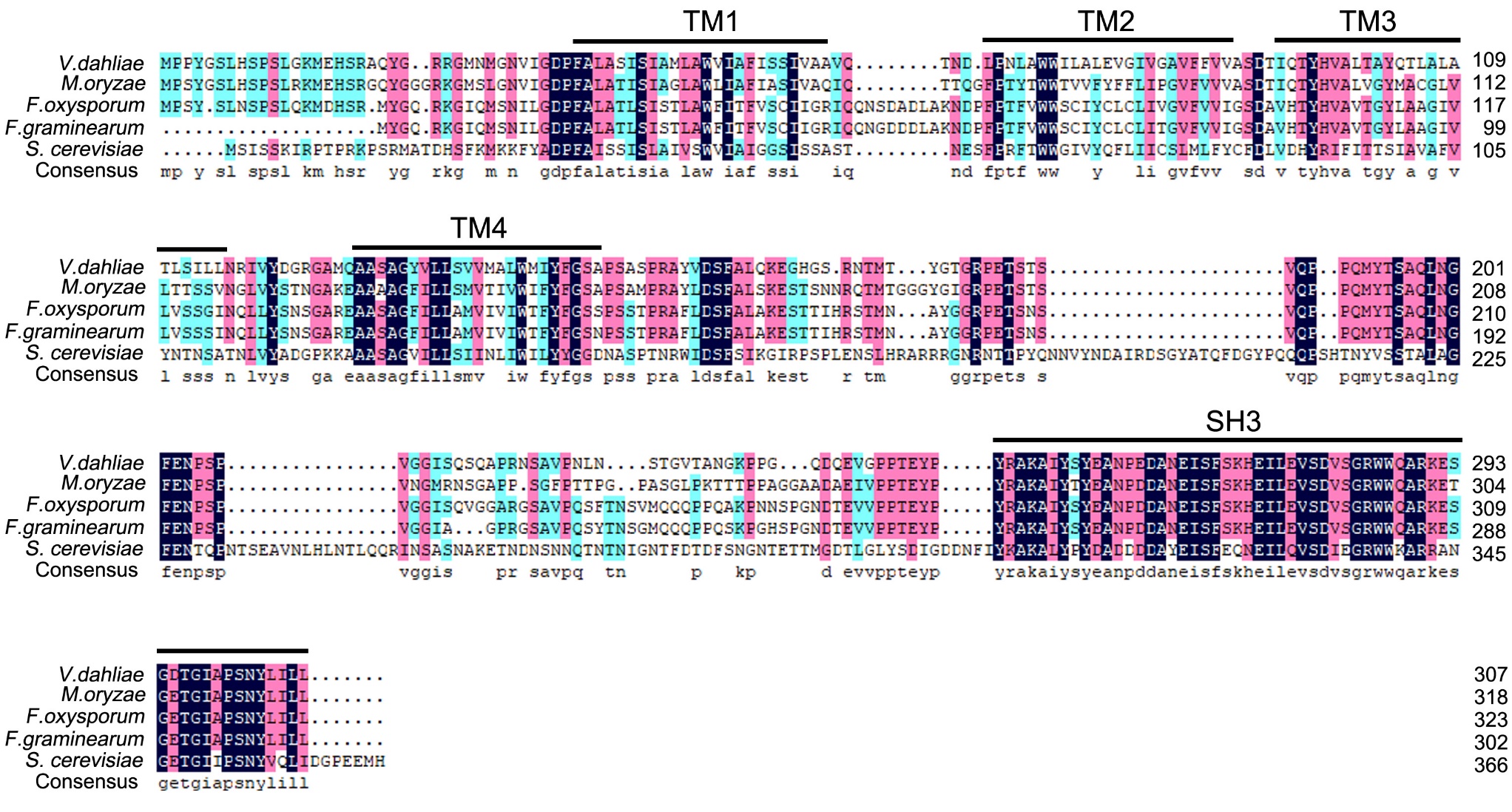
**

**Figure S2**


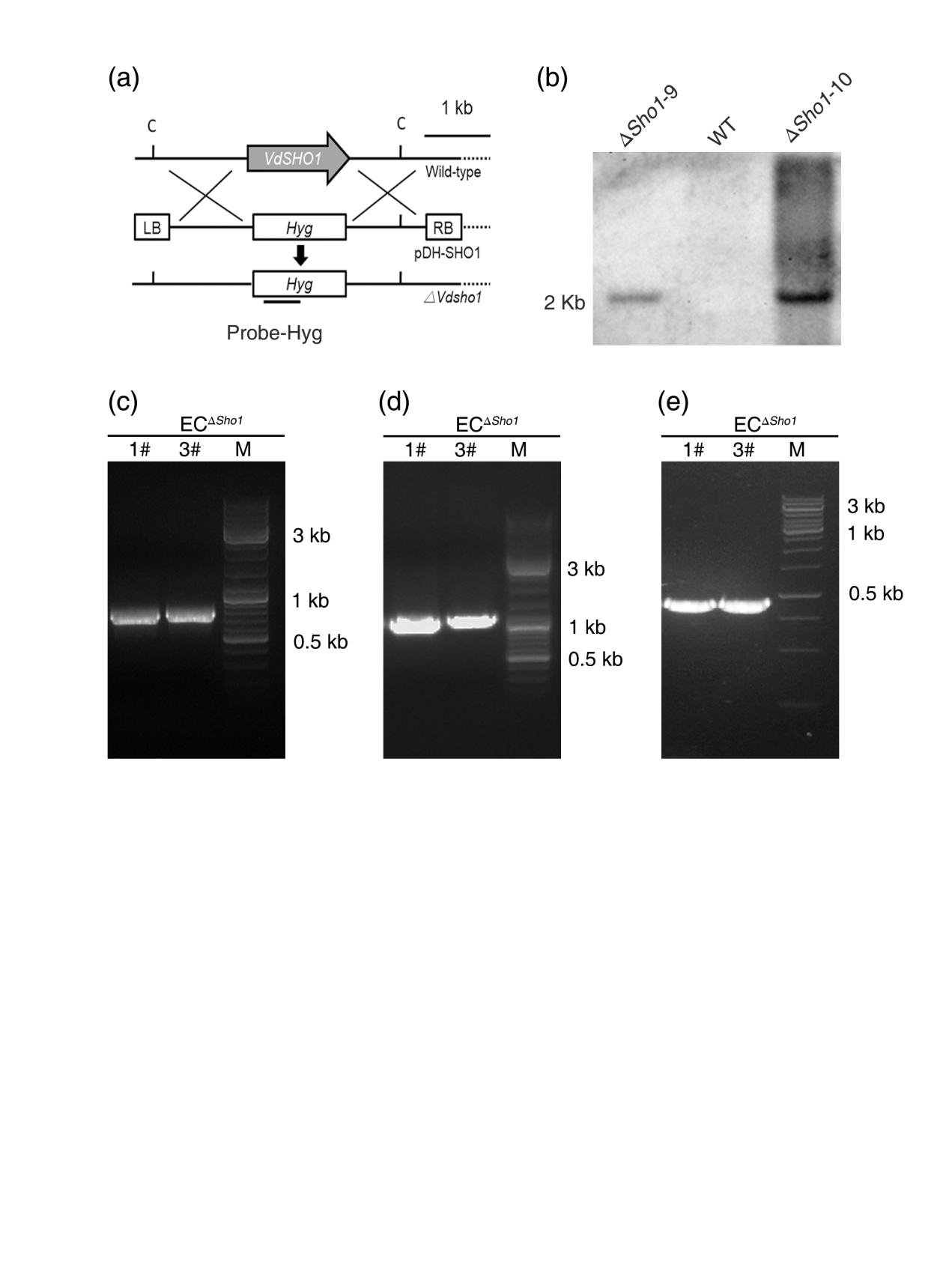


**Figure S3**


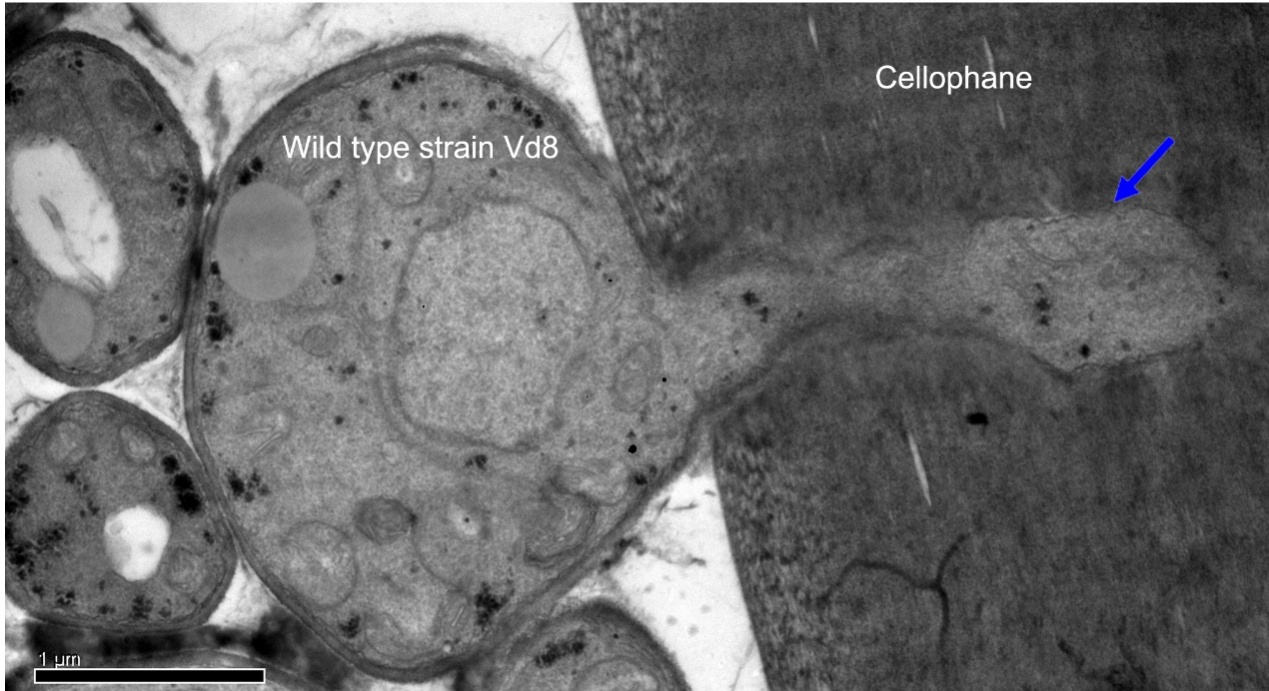


**Figure S4**


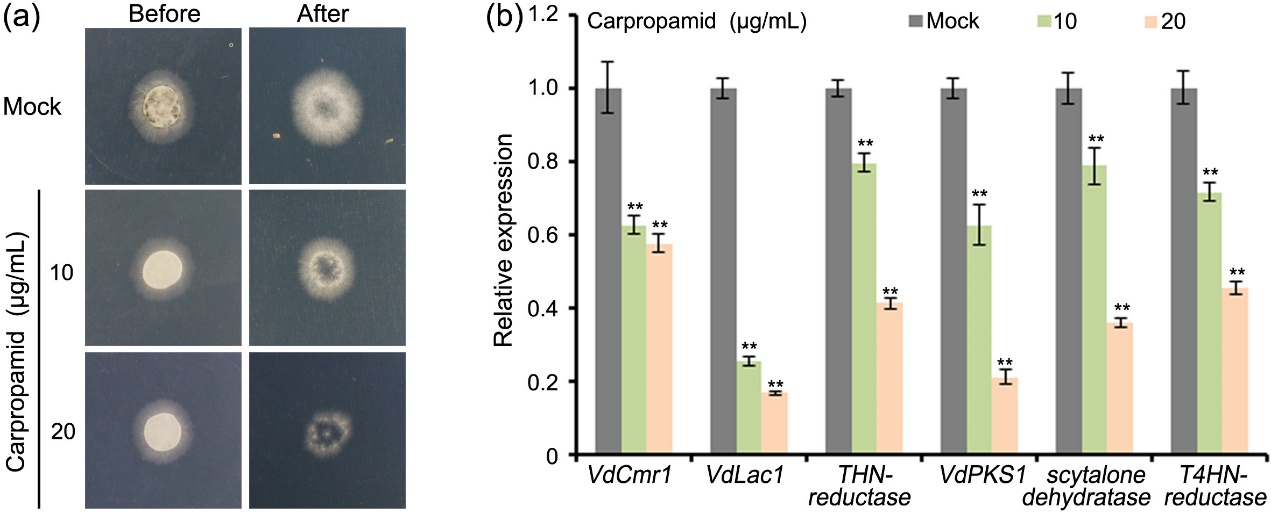


**Figure S5**


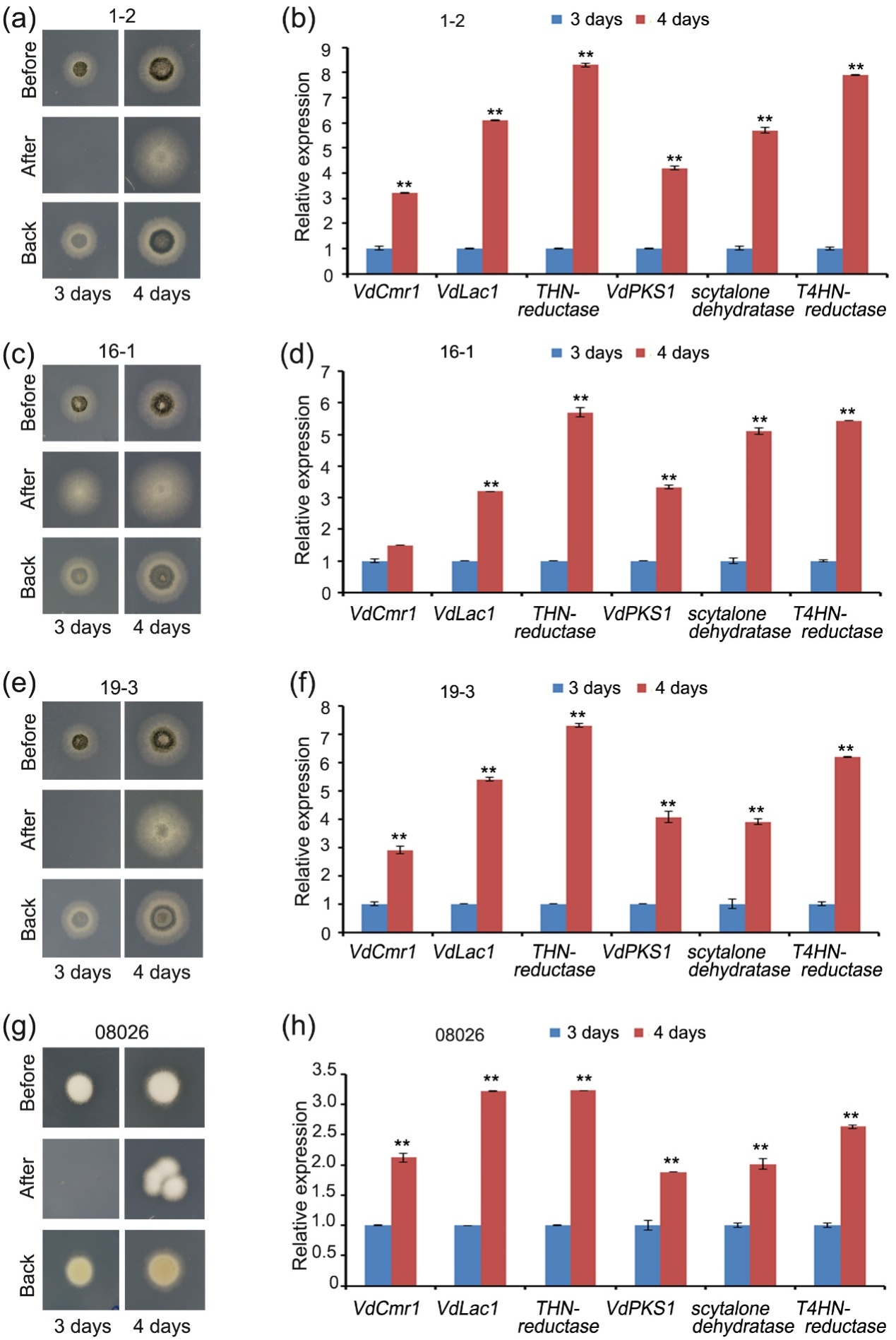


**Figure S6**

**
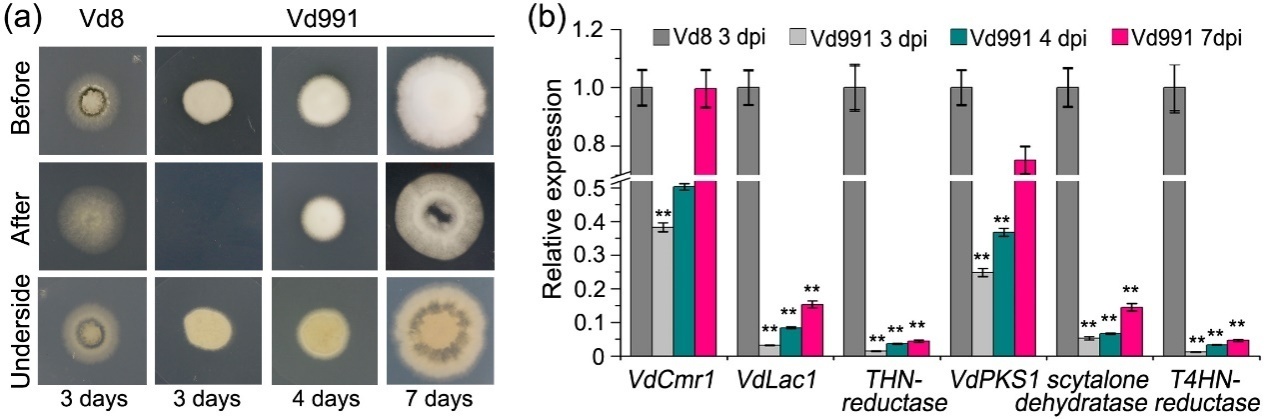
**

**Figure S7**

**
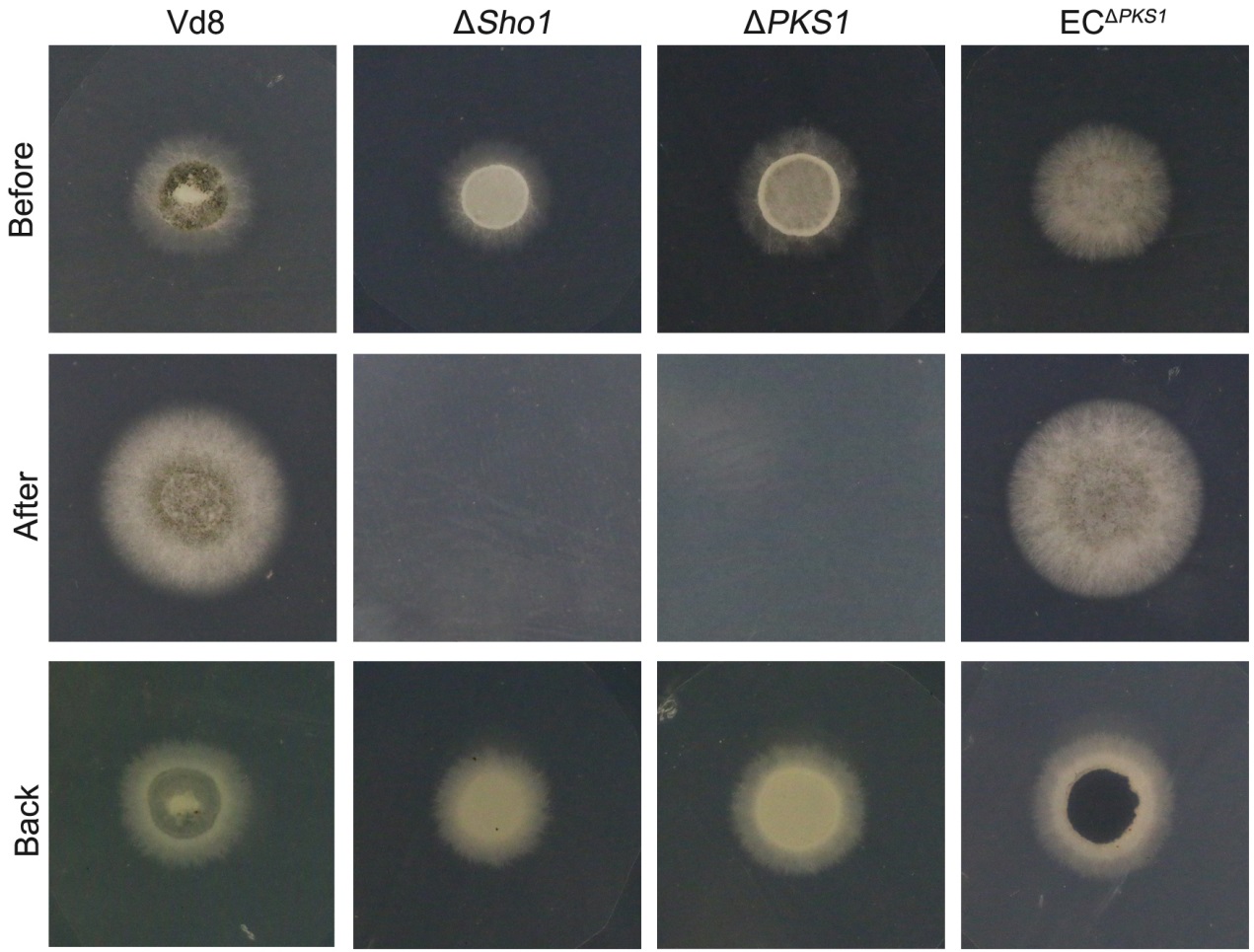
**

**Figure S8**

**
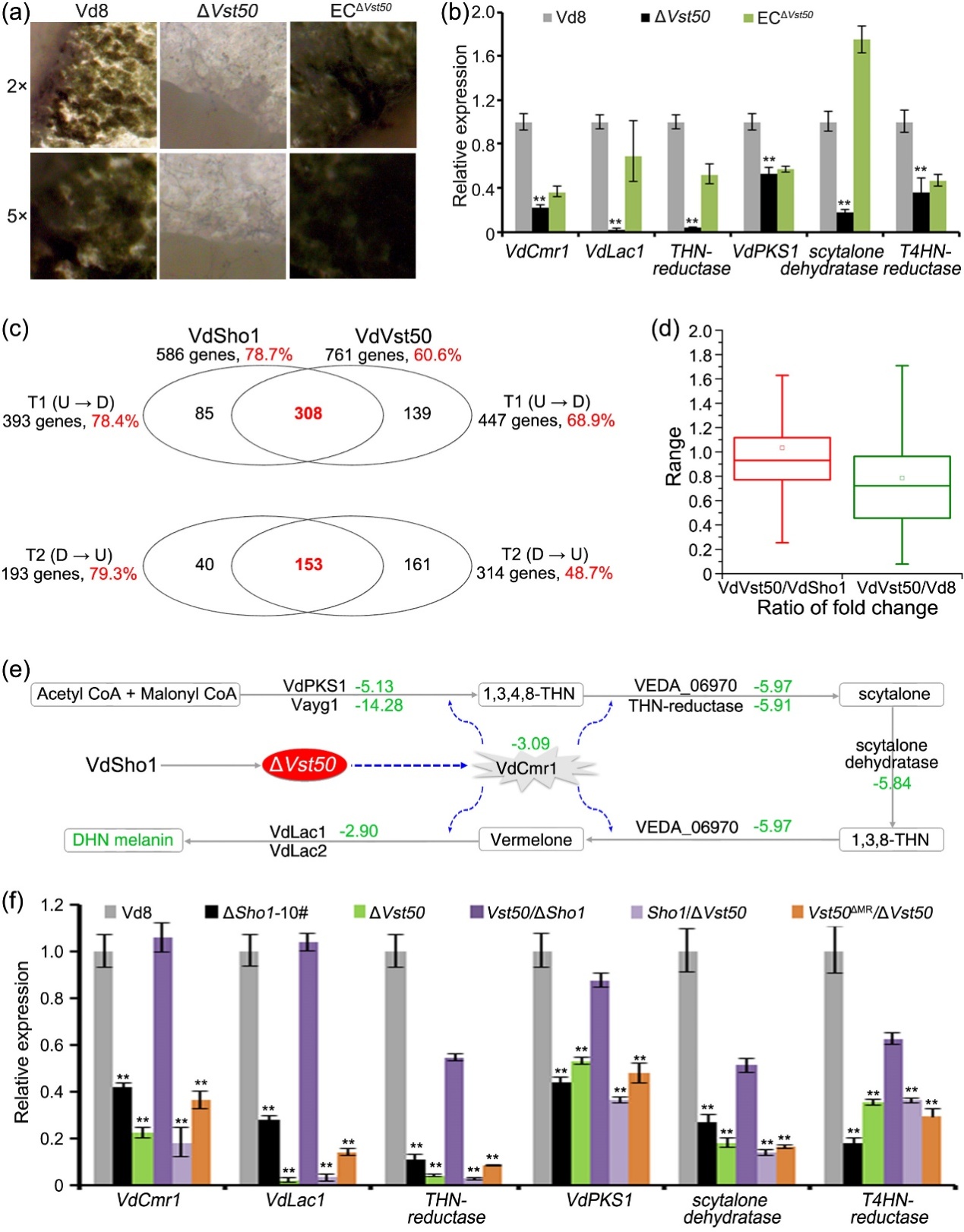
**

**Figure S9**


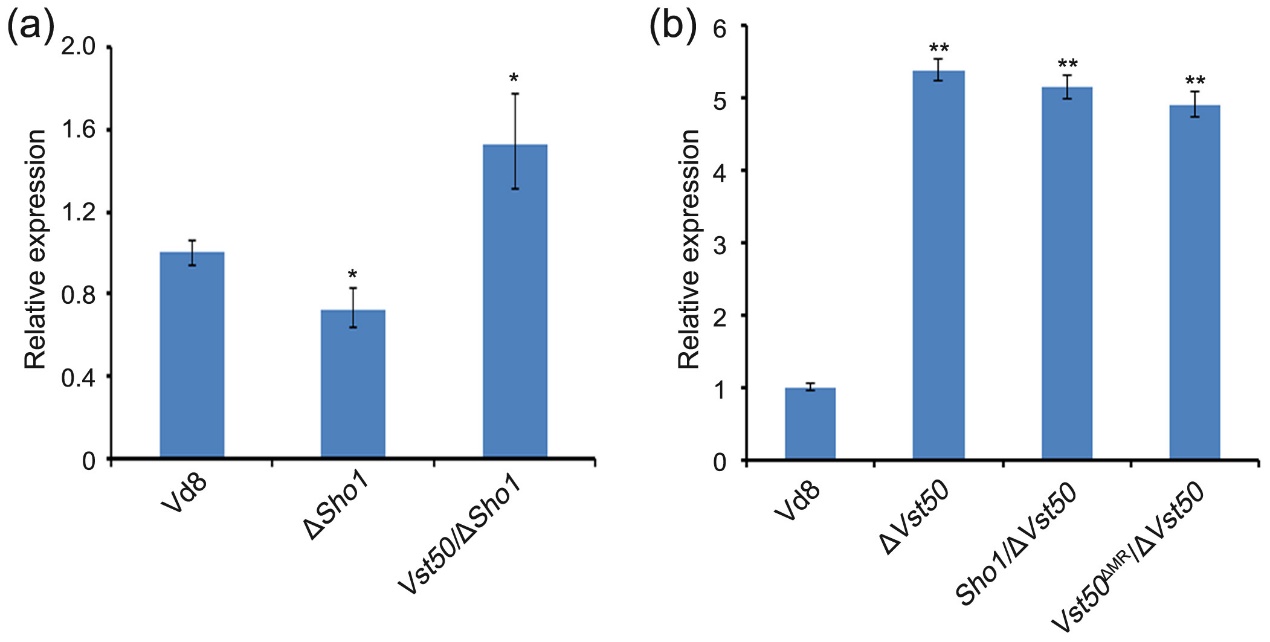


**Figure S10**

**
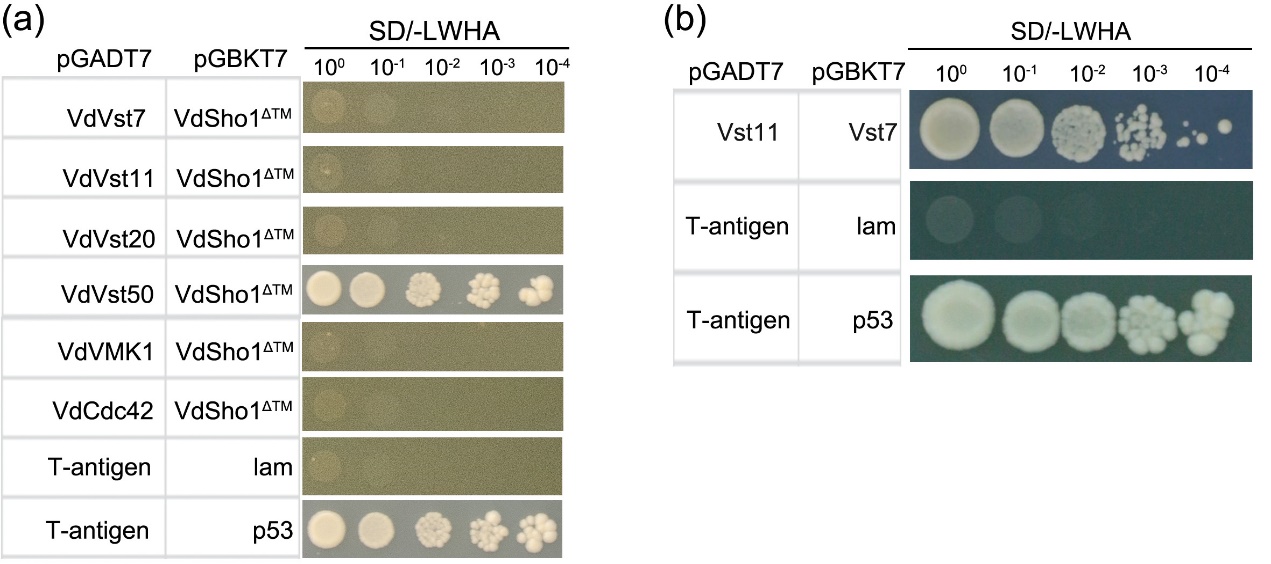
**

**Figure S11**

**
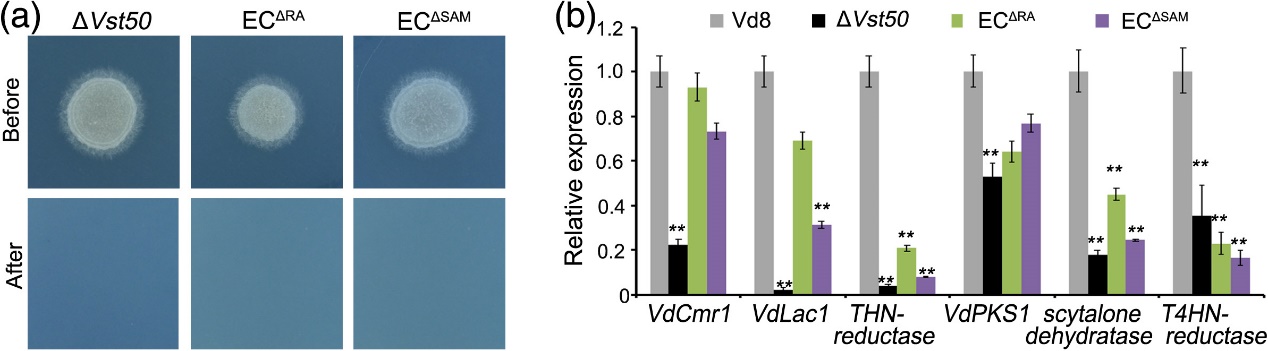
**

**Figure S12**


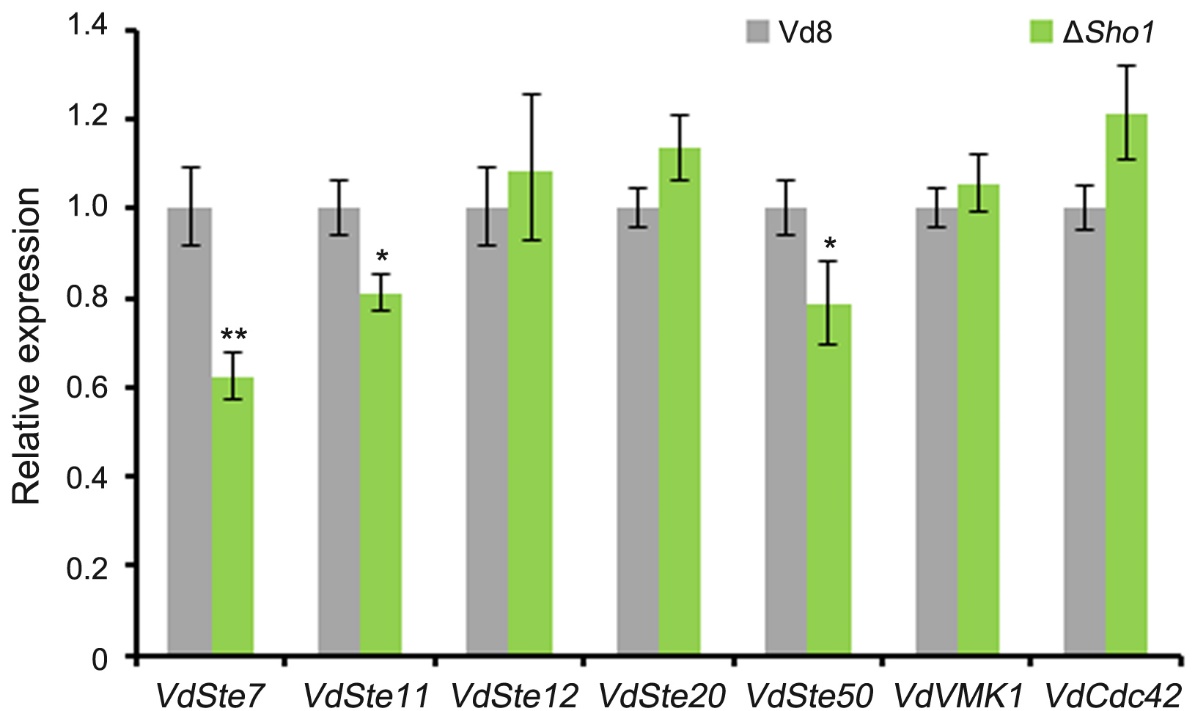


**Figure S13**

**
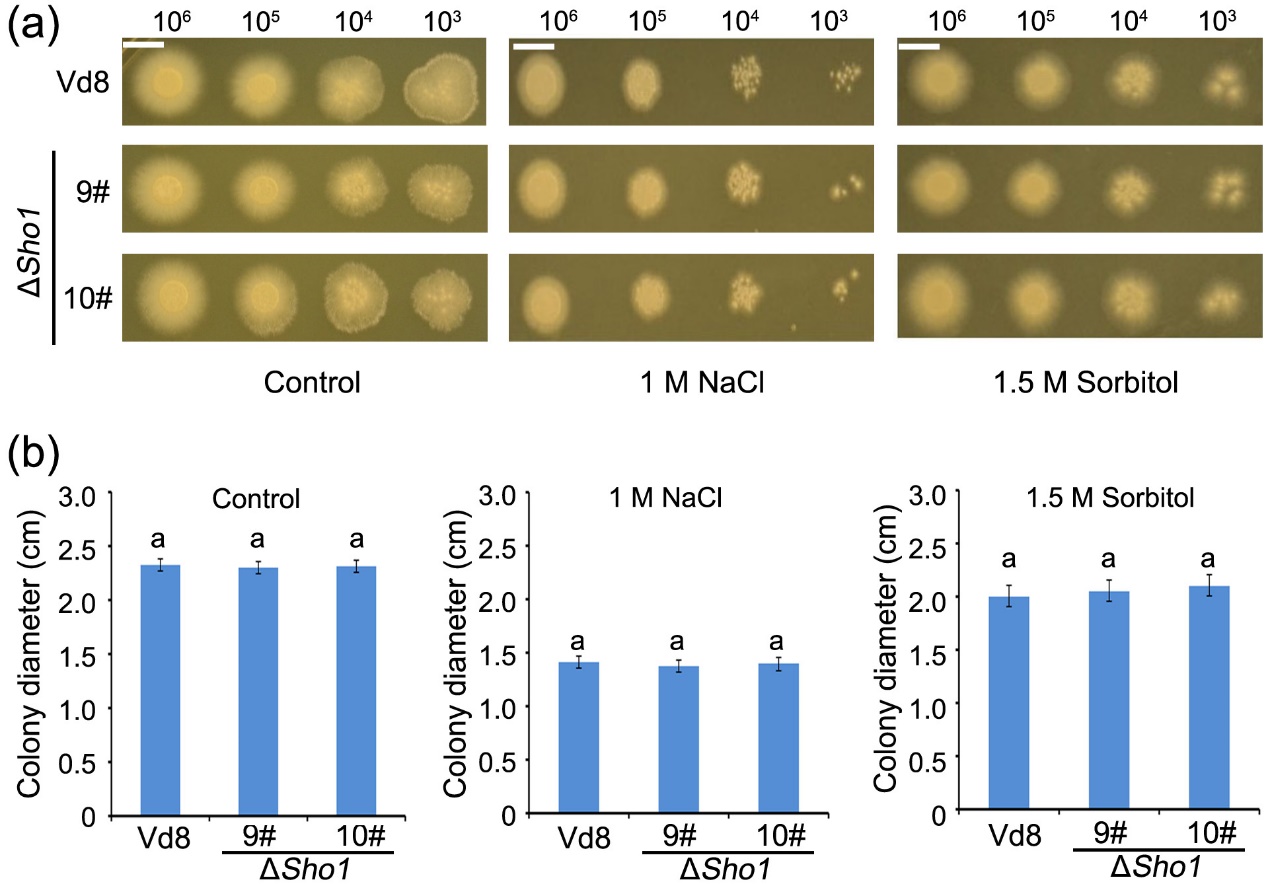
**

**Figure S14**

**
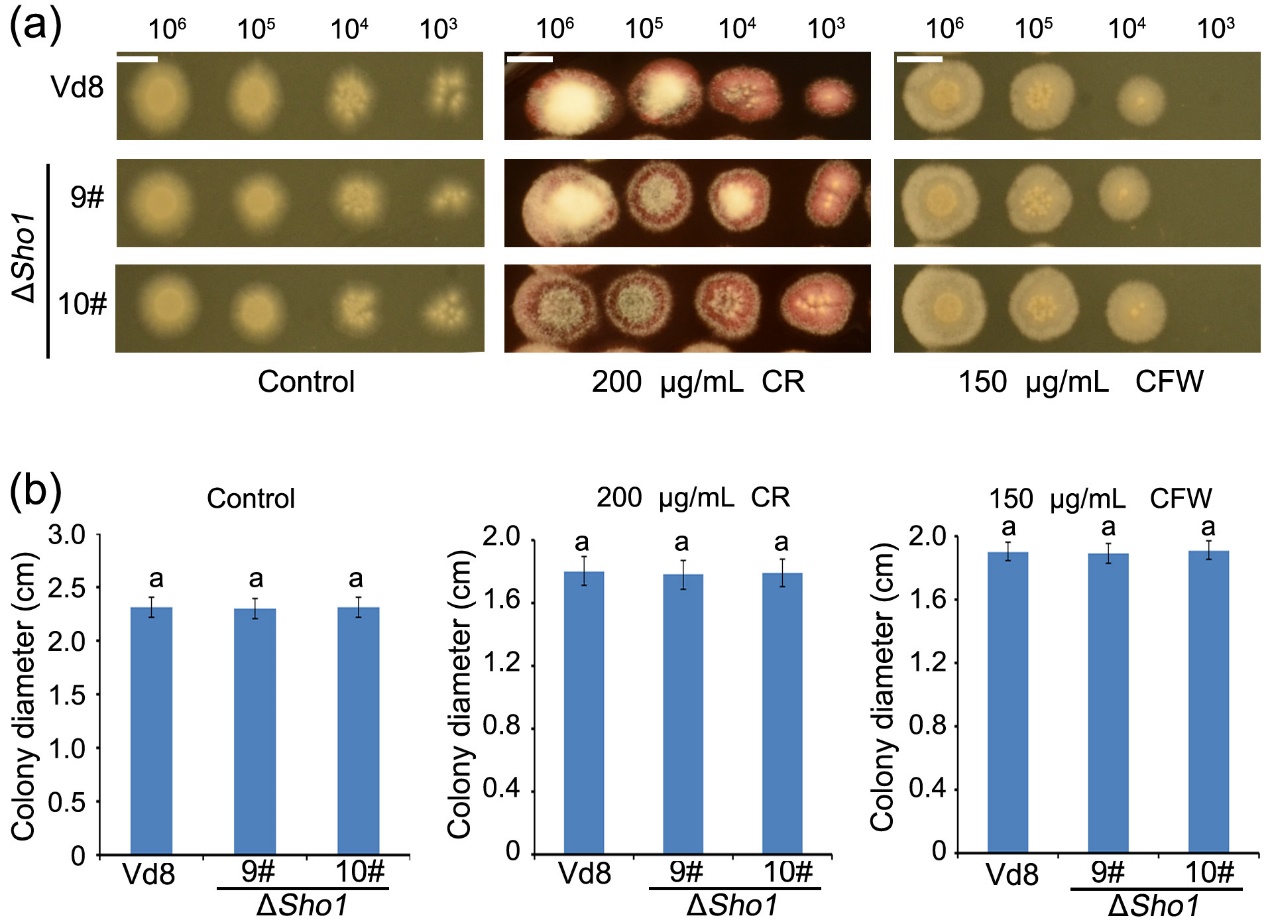
**

**Figure S15**

**
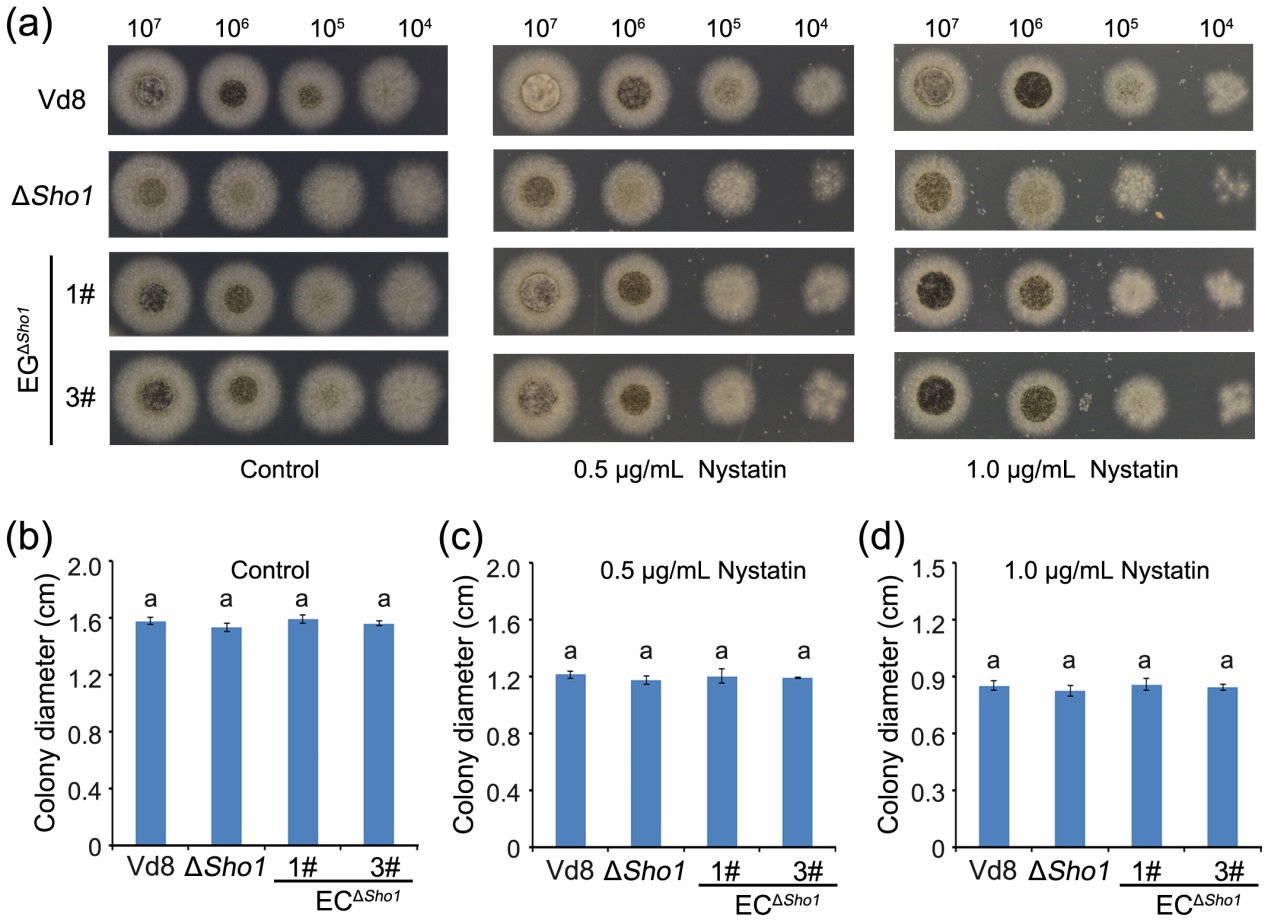
**

**Figure S16**


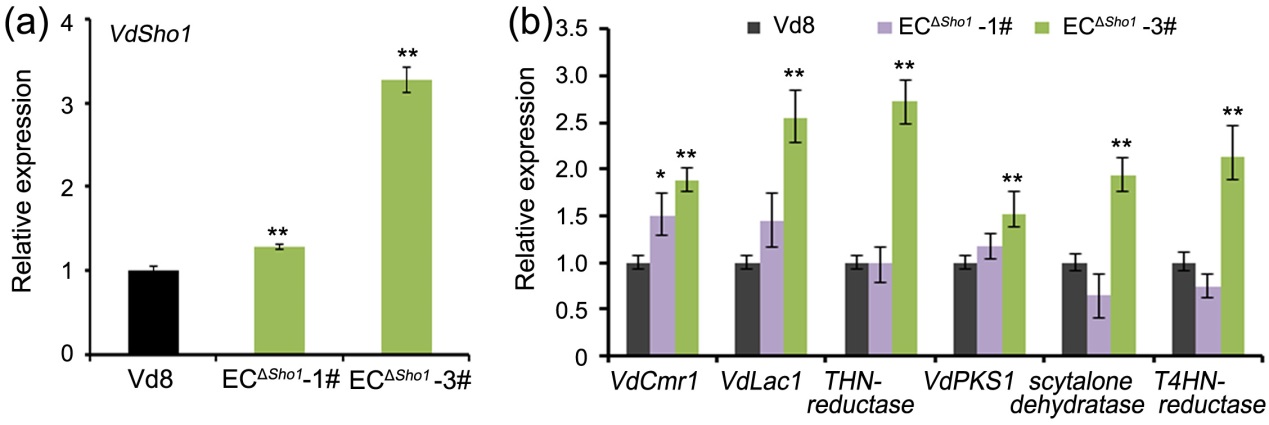


**Figure S17**

**
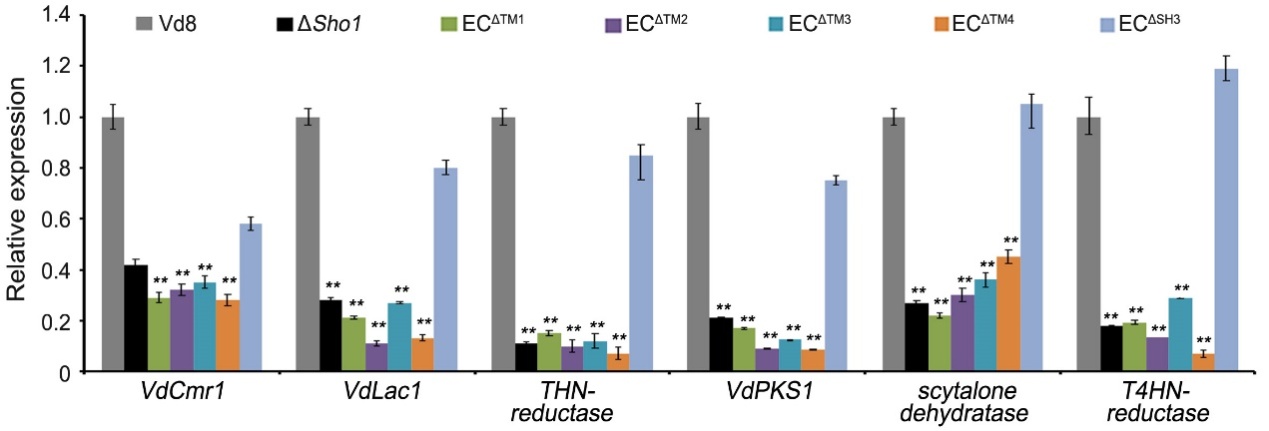
**

**Figure S18**

**
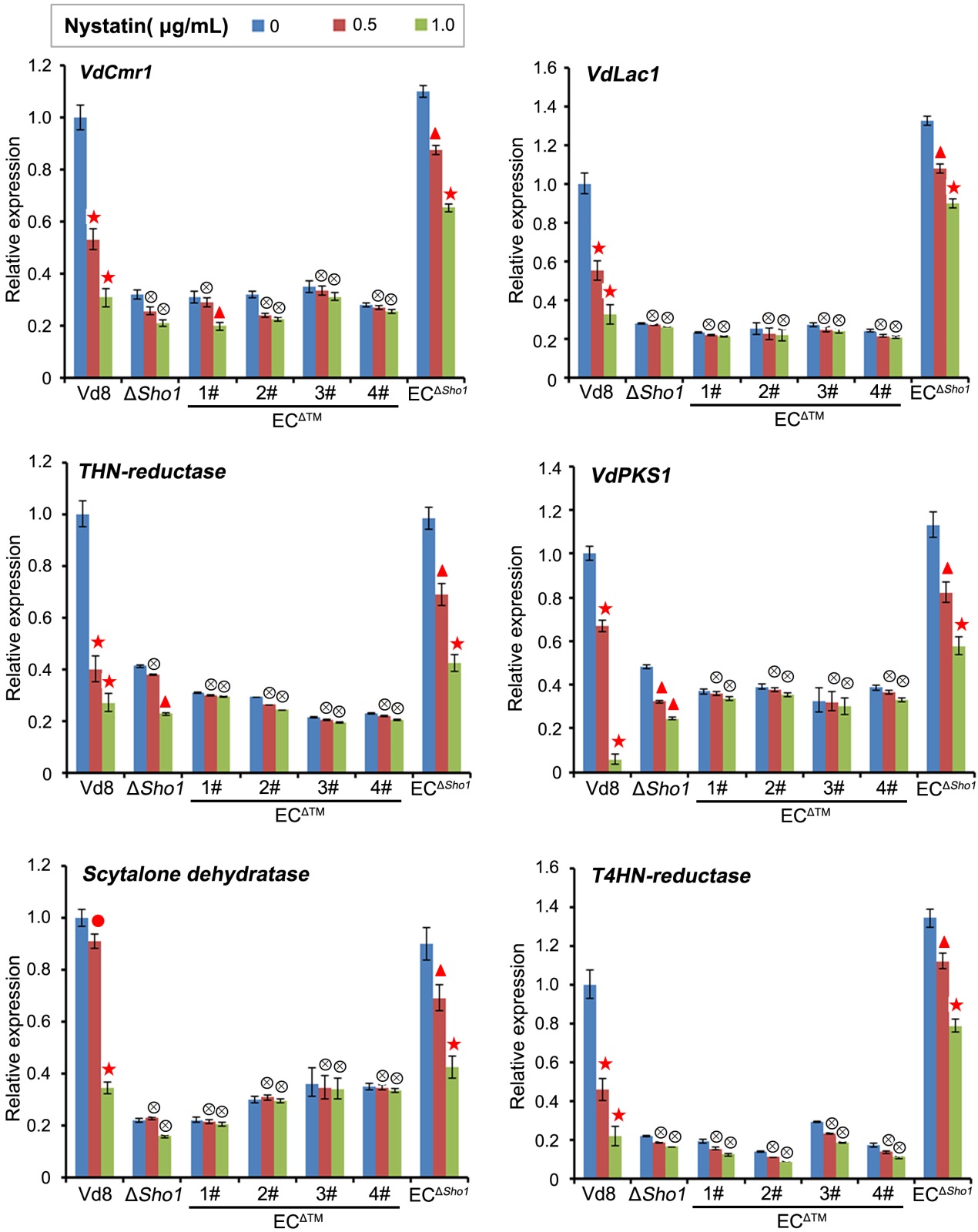
**

**Figure S19**

**
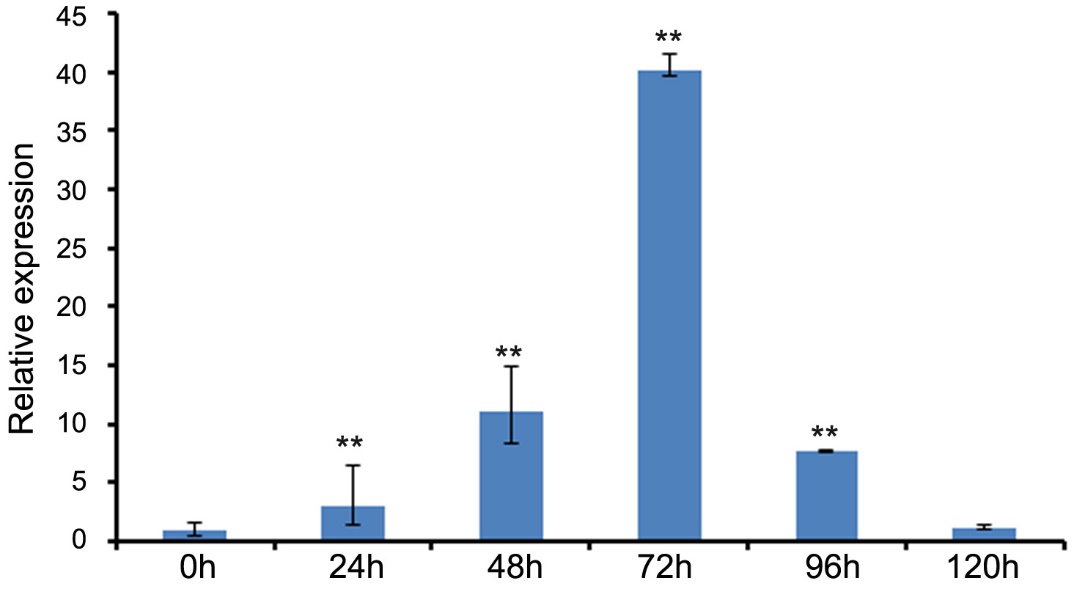
**

**Figure S20**

**
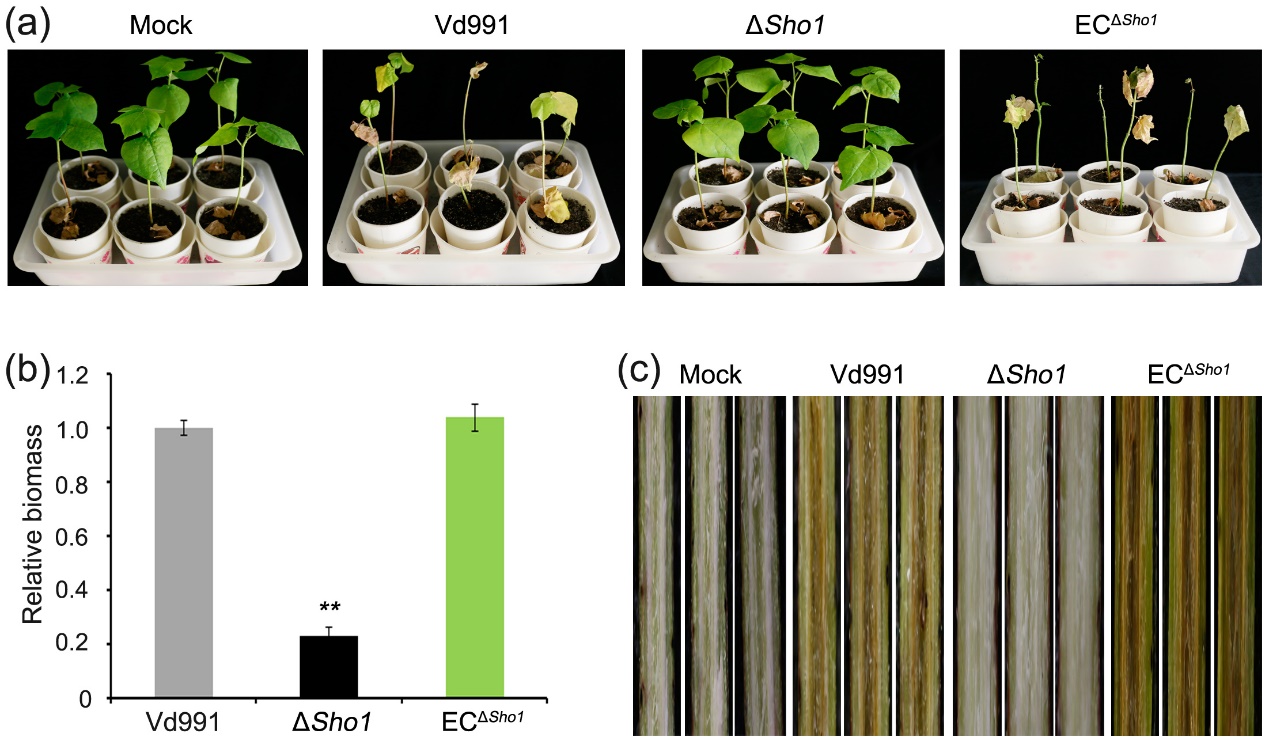
**

**Figure S21**

**
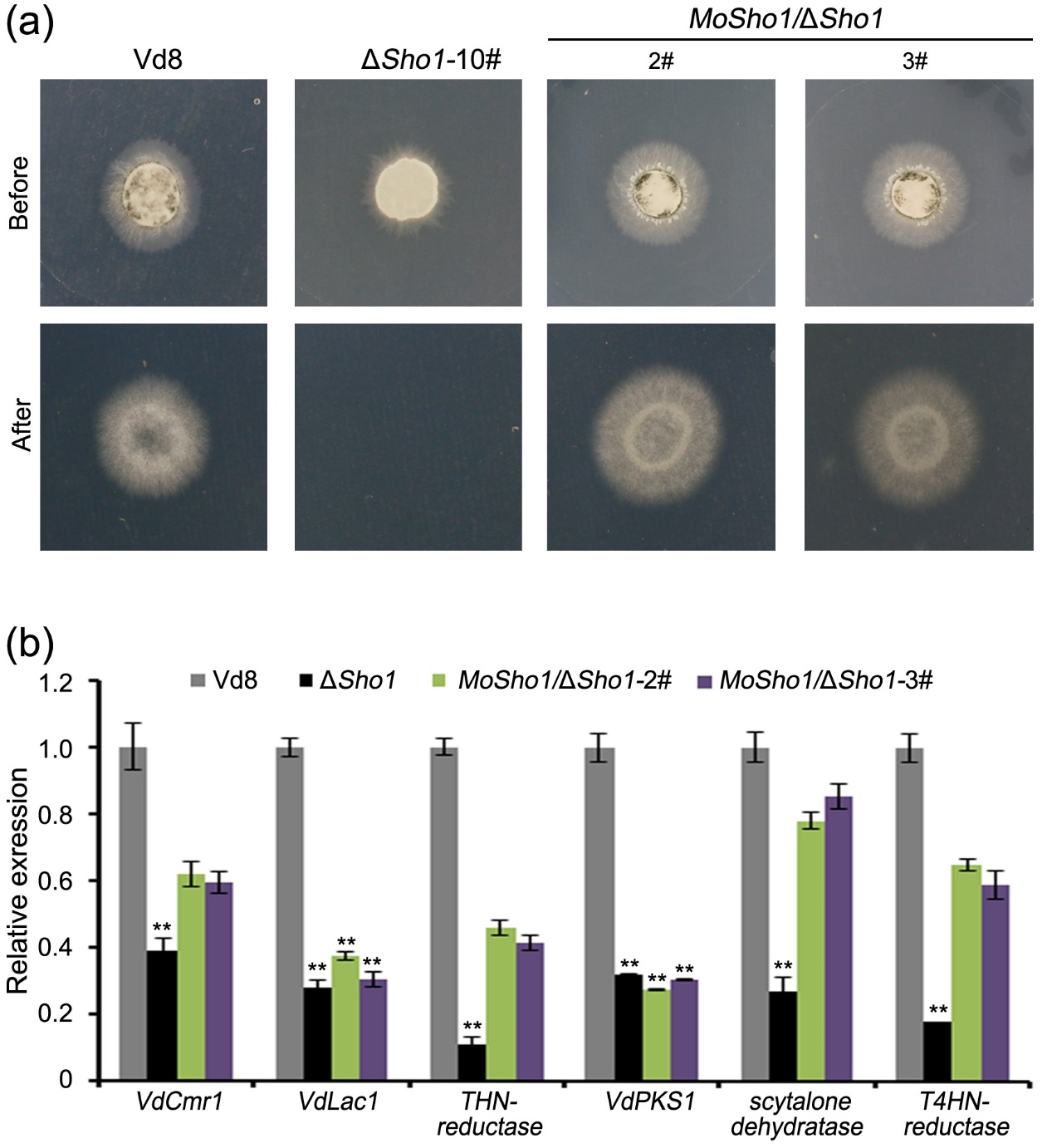
**

**Figure S22**

**
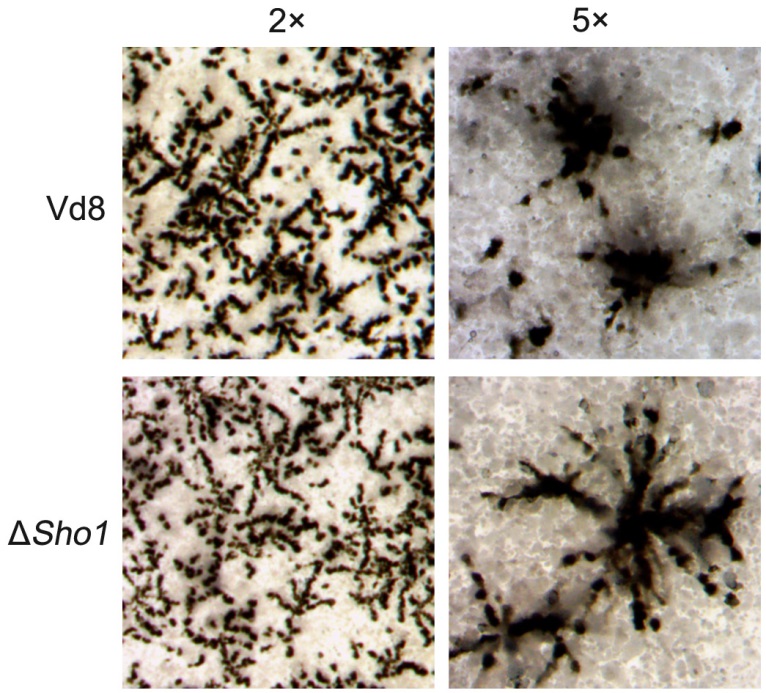
**
